# Synthesis, Biological Evaluation and Molecular Docking of Novel Indole-Aminoquinazoline Hybrids for Anticancer Properties

**DOI:** 10.3390/ijms19082232

**Published:** 2018-07-31

**Authors:** Malose J. Mphahlele, Mmakwena M. Mmonwa, Abimbola Aro, Lyndy J. McGaw, Yee Siew Choong

**Affiliations:** 1 Department of Chemistry, University of South Africa, Private Bag X06, Florida 1710, South Africa; mmonwmm@unisa.ac.za; 2 Phytomedicine Programme, Department of Paraclinical Sciences, University of Pretoria, Private Bag X04, Onderstepoort 0110, South Africa; titotani2014@gmail.com (A.A.); lyndy.mcgaw@up.ac.za (L.J.M.); 3 Institute for Research in Molecular Medicine (INFORMM), Universiti Sains Malaysia, 11800 Minden, Penang 11800, Malaysia; yeesiew@usm.my

**Keywords:** indole-aminoquinazolines, cytotoxicity, apoptosis, EGFR-TK, molecular docking

## Abstract

A series of indole-aminoquinazolines was prepared via amination of the 2-aryl-4-chloroquinazolines with the 7-amino-2-aryl-5-bromoindoles. It was then evaluated for cytotoxicity in vitro against human lung cancer (A549), epithelial colorectal adenocarcinoma (Caco-2), hepatocellular carcinoma (C3A), breast adenocarcinoma (MCF-7), and cervical cancer (HeLa) cells. A combination on the quinazoline and indole moieties of a 2-phenyl and 2-(4-fluorophenyl) rings in compound **4b**; 2-(4-fluorophenyl) and 3-chlorophenyl rings in compound **4f**; or the two 2-(4-fluorophenyl) rings in compound **4g**, resulted in significant and moderate activity against the Caco-2 and C3A cell lines. The indole-aminoquinazoline hybrids compounds **4f** and **4g** induced apoptosis in Caco-2 and C3A cells, and were also found to exhibit moderate (IC_50_ = 52.5 nM) and significant (IC_50_ = 40.7 nM) inhibitory activity towards epidermal growth factor receptor (EGFR) against gefitinib (IC_50_ = 38.9 nM). Molecular docking suggests that 4a–h could bind to the ATP region of EGFR like erlotinib.

## 1. Introduction

**Figure 1:**
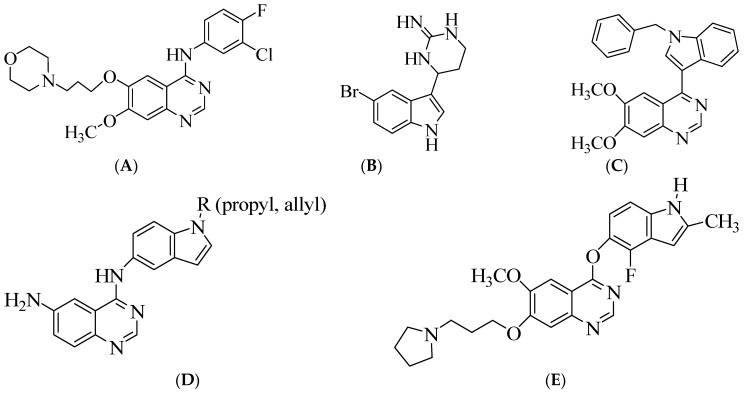
Structure of gefitinib (**A**), aplicyanin A (**B**) and indole-appended quinazoline hybrids (**C**–**E**) of biological importance.

Nitrogen-containing heterocycles, such as quinazolines and indoles, have earned great interest in targeted therapies as antitumor drugs. The 4-anilinoquinazolines, such as the clinical drug gefitinib shown in Figure 1A, for example, are known to produce their anticancer activity through inhibition of the epidermal growth factor receptor tyrosine kinase (EGFR-TK) phosphorylation—which results from competitive binding at the ATP site [1,2,3]. The indole nucleus, on the other hand, is prevalent in many heterocycles with antimicrobial, antioxidant, anticancer and anti-tubercular activities [4,5,6,7]. A lipophilic bromine atom on the fused benzo ring of an indole framework has been found to impart significant anti-tumour activity in both synthetic [8] and naturally [9] occurring indoles, such as aplicyanin A (Figure 1B), shown in Figure 1. The latter exhibits increased antiproliferative activity against the human breast cancer (MDA-MB-231), lung cancer (A549) and colon cancer (HT-29) cell lines [9]. 4-(1-Benzyl-1*H*-indol-3-yl)-6,7-dimethoxyquinazoline (Figure 1C) is an example of an indole-quinazoline hybrid that was previously found to exhibit moderate ErbB-2 activity, with little or no activity against the epidermal growth factor receptor [10]. The analogous 4-(indole-3-yl)quinazolines were found to be highly potent EGFR-TK inhibitors with excellent cytotoxic properties against several cancer cell lines [11]. Significant anti-inflammatory properties and dose-dependent lipopolysaccharide-induced TNF-α and IL-6 release was observed for the *N*^4^-(1*H*-indol-5-yl)quinazoline-4,6-diamines (Figure 1D) [12]. Cediranib (AZD2171) shown in Figure 1E, on the other hand, is an example of an *N*-unsubstituted indole-ether quinazoline hybrid that has been found to be a highly potent, orally bioavailable and selective vascular endothelial growth factor receptor (VEGFR) inhibitor for the treatment of cancer [13,14]. We considered our previous work on the antiproliferative properties of the 2-arylindoles [15] and the 4-anilinoquinazolines [16,17] in combination with the literature analyses on bioactive compounds containing these moieties and decided to merge the two pharmacophores to comprise the indole-aminoquinazoline analogues of cediranib (Figure 1E).

Since most of the primary 4-aminoquinazolines only differ in terms of the substituents and side chains, we decided to replace the aniline group of the 2-arylquinazolines with a 2-arylindole moiety to comprise the indole-appended 4-aminoquinazoline analogous of cediranib (Figure 1E). The main aims were to evaluate the resultant hybrids for antigrowth effect against a panel of cancer cell lines. These include; human lung cancer (A549), epithelial colorectal adenocarcinoma (Caco-2), hepatocellular carcinoma (C3A; HepG2/C3A), breast adenocarcinoma (MCF-7), and cervical (HeLa) cancer cell lines. Since cytotoxicity does not define the mode of cancer cell death, we evaluated the most cytotoxic compounds for their ability to induce apoptosis. Moreover, the most active compounds were evaluated for their capability to inhibit EGFR-TK phosphorylation complemented with molecular docking (in silico) into the ATP binding site of EGFR.

## 2. Results and Discussion

### 2.1. Chemistry

**Scheme 1:**
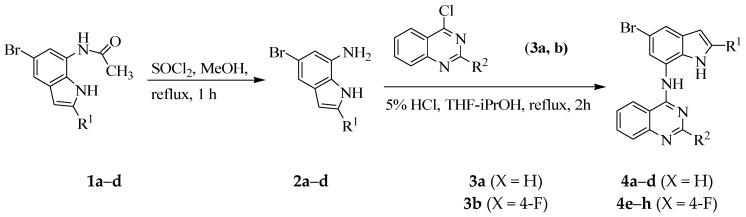
Synthesis of indole-aminoquinazolines compounds **4a**–**h**.

The synthesis of the requisite indole-aminoquinazolines involved the amination of the 2-aryl-4-chloroquinaolines with the known 7-acetamido-2-aryl-5-bromoindoles, compounds **1a**–**d** [18] as represented in Scheme 1 and Table 1. The ^1^H- and ^13^C-NMR spectra of compounds **2a–d** and **4a–h** are included as Figure S1 in the Supplementary Materials. In order to prepare the 7-amino-2-aryl-5-bromoindoles **2a**–**d** to serve as substrates for the amination of the 4-chloroquinazolines, compounds **3a** or **3b**, we subjected the acetamido derivatives **1a**–**d** to hydrolysis with thionyl chloride in methanol under reflux for 1 h. We isolated by aqueous work-up and column chromatography on silica gel products, characterised by means of nuclear magnetic resonance (^1^H-NMR and ^13^C-NMR) and infrared (IR) spectroscopic techniques, complemented with mass spectrometry as the requisite 7-amino-2-aryl-5-bromoindoles, **2a**–**d**. These compounds were easily distinguished from the corresponding precursors by the absence of signals corresponding to the acetyl group in their ^1^H- and ^13^C-NMR spectra. Previously, the analogous 2,5- and 3,5-disubstituted 7-aminoindoles have been prepared via a three component Wittig reaction of pyrrole-3-carboxaldehydes with fumaronitrile and PEt_3_, followed by chemoselective C-6 alkylation of the incipient *cis*-allylic nitriles, and subsequent cyclization through an intramolecular Houben−Hoesch reaction [19]. Cacchi et al. [20] have also developed an alternative method for the synthesis of the 7-aminoindole derivatives, which involves the Buchwald-Hartwig C–N bond formation of the intermediate 7-bromoindoles with primary and secondary amines. The 2-aryl-4-chloroquinazolines compounds, **3a** and **3b**, used as coupling partners, were prepared by treatment of the known 2-arylquinazolin-4(3*H*)-ones with phosphoryl chloride under reflux, following the literature procedure [21]. Amination of the electrophilic 4-chloroquinazoline derivatives, **3a** (X = H) and **3b** (X = 4-F), with 7-aminoindoles, **2a**–**d**, in the presence of 5% HCl in tetrahydrofuran-isopropanol (THF-iPrOH) mixture under reflux afforded the corresponding indole-aminoquinazoline hybrids, **4a**–**h**. Their ^1^H-NMR spectra revealed the presence of increased signals in the aromatic region and two singlets integrating for single proton each around δ 11.4 and 12.0 ppm, which correspond to the endocyclic nitrogen and the nitrogen bridge (4-amino group), respectively. The calculated *m*/*z* values for the molecular hybrids, **4a**–**h**, were found to be consistent with the molecular ions of the assigned structures.

As an introduction to indole-appended 4-aminoquinazoline hybrids with potential anticancer activity we decided to evaluate compounds **4a**–**h** for in vitro antigrowth effect against: Human lung cancer (A549), epithelial colorectal adenocarcinoma (Caco-2), hepatocellular carcinoma (C3A; HepG2/C3A), breast adenocarcinoma (MCF-7), and cervical (HeLa) cell lines. The structure activity relationship (SAR) of these compounds has been rationalised with respect to the substitution patterns on the phenyl rings of the quinazoline and indole components.

### 2.2. Biological Evaluation

#### 2.2.1. In Vitro Cytotoxicity of Indole-Aminoquinazoline Hybrids **4a**–**h**

Compounds **4a**–**h** were evaluated for potential antiproliferative effect against the A549, Caco-2 and C3A (HepG2/C3A), MCF-7, and HeLa cell lines using the well-established 3-(4,5-dimethylthiazole-2-yl)-2,5-diphenyltetrazoliumbromide based colorimetric cell viability (MTT) assay. The compounds were assayed as dimethyl sulfoxide (DMSO) solutions at the following concentrations: 5, 12.5, 25, 50, 100 μM, against gefitinib as a reference standard. DMSO was used as a negative control of choice since it has been found before to have no apparent effect on proliferation of the HeLa, Caco-2, and C3A cells at 1% or less for up to 48 h [22,23]. Gefitinib used as a positive control, on the other hand, has previously been found to induce autophagy in lung cancer A549 cells and to enhance apoptosis and suppress cancer cell proliferation [2]. Moreover, the Caco-2 cells have been found to display greater sensitivity to gefitinib due to their high EGFR expression [24]. The cytotoxic activities of the tested compounds were expressed as LC_50_ values (the dose that reduces survival to 50%) in μM concentrations, using DMSO as the negative control and gefitinib as a positive control. The LC_50_ values of the tested compounds (average from three independent experiments) against gefitinib as reference drug are represented in Table 2. Gefitinib resulted in different degree of cell growth inhibition for the A549 (modest), Caco-2 (intermediate), C3A (highly sensitive), MCF-7 (moderate) cell lines, with no inhibition of cell growth for the HeLa cells. Within the series **4a**–**d**, compound **4a** substituted with a phenyl ring on both the quinazoline and indole moiety was found to be inactive against all the cancer cell lines tested. The presence of a phenyl group on the quinazoline ring and a small lipophilic fluorine atom at the 4-position of the phenyl ring of the indole moiety in **4b** resulted in significant cytotoxicity against all the five cancer cell lines. Compound **4c** substituted with a chlorine atom at the 3-position of the phenyl ring of the indole moiety and a phenyl ring at position-2 of the quinazoline scaffold was found to exhibit significant cytotoxicity against A549 and Caco-2 when compared to gefitinib. Modest cytotoxicity was observed for this compound against the C3A, MCF-7, and HeLa cell lines. The presence of a bulky 4-methoxyphenyl ring at the C-2 position of the indole framework of **4d** resulted in diminished activity against all the cancer cell lines tested. Compound **4e** substituted with a 2-phenyl ring on the indole moiety and a 4-fluorophenyl group at position-2 of the quinazoline framework was found to be inactive against the Caco-2, C3A, MCF-7 and HeLa cells, but to exhibit cytotoxicity against the A549 cells (LC_50_ = 51.64 µM) comparable to the reference standard (LC_50_ = 51.29 µM). The presence of the 4-fluorophenyl group at the C-2 positions of both quinazoline and indole moieties in compound **4f**, on the other hand, also resulted in significant cytotoxicity against the four cancer cell lines. The observed cytotoxicity of 4b and 4f is presumably due to the increased lipophilicity of the molecule resulting from the non-polarizability of the C*sp*^2^-F bond [25]. Moreover, fluorine substitution is known to improve the overall pharmacokinetics and pharmacodynamics of the molecule by improving solubility, selectivity, bioavailability and metabolic stability [26]. Within the series **4e**–**h**, compound **4g** substituted with a 3-chlorophenyl group on the indole moiety and a 4-fluorophenyl group at the 2-position of the quinazoline moiety was found to be more cytotoxic against the Caco-2 (LC_50_ = 6.46 µM) cell line when compared to gefitinib (LC_50_ = 27.91 µM). It has previously been observed for the analogous halogenated 4-anilinoquinazolines, which were identified as non-competitive antagonists of metabotropic glutamate receptor 5 (mGlu_5_) that a small lipophilic group (X = F, Cl, Br) at the 3-position of the aniline ring resulted in up to 10-fold increase in potency [27]. Compound **4g** was also found to exhibit moderate cytotoxicity against the C3A (LC_50_ = 12.20 µM), MCF-7 (LC_50_ = 39.07 µM) and HeLa (LC_50_ = 25.51 µM) cells. A combination of the bulky 4-methoxyphenyl group on the indole framework with a 4-fluorophenyl group on the quinazoline component in 4h resulted in moderate cytotoxicity against the A549 and Caco-2 cell lines and reduced activity against the C3A cell line. Significant cytotoxicity for compounds **4b**, **4f** and **4g** and moderate activity observed for 4c against the CaCo-2 and C3A cells may suggest the presence of a halogen atom on the phenyl ring of the indole moiety to be essential for the antiproliferative properties of these indole-aminoquinazolines.

Gefitinib has been found to inhibit tumour pathogenesis, metastasis and angiogenesis, and it also promotes apoptosis [3]. Since cytotoxicity does not define a specific cellular death mechanism (apoptosis vs. necrosis) and considering the pro-apoptotic properties of the 4-anilinoquinazolines [3,28] and indole-3-carbinol [29,30], we decided to evaluate how compounds **4a**–**h** induce cell death in these cancer cells.

#### 2.2.2. Evaluation of Cell Death Pathways

**Figure 2:**
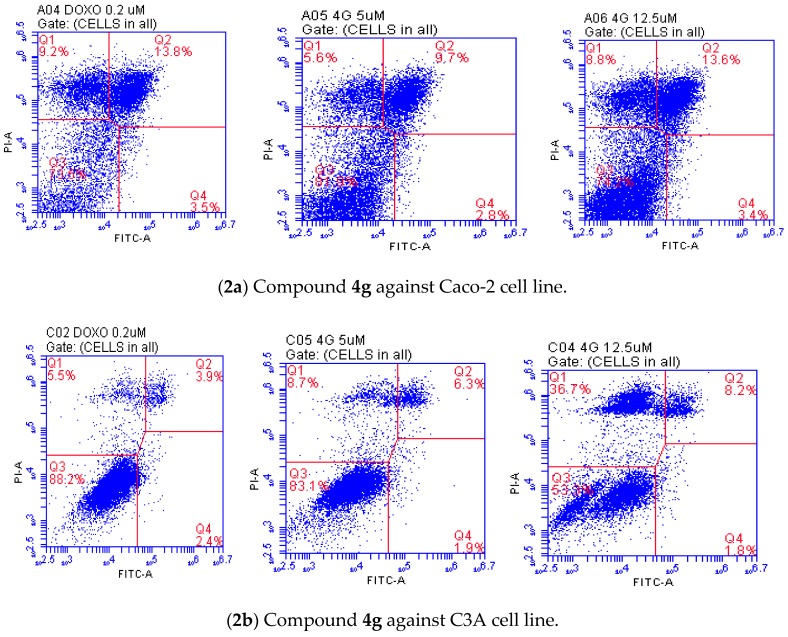
Effects of compound **4g** on the induction of apoptosis in the Caco-2 (2a) and C3A cells (2b) at 5 and 12.5 µM after 24 h against doxorubicin hydrochloride as determined by Annexin V/PI staining.

Apoptosis is one of essential mechanisms through which the body is able to get rid of unwanted cells, and triggering this process in cancer cells would lead to automatic death and decrease cancer proliferation. Necrosis, on the other hand, refers to changes following cell death by any mechanism including apoptosis, and it does not necessarily tell how cell death occurred because it takes place after the cells have already died, and reached equilibrium with their surroundings [31,32]. We selected compound **4g** to evaluate whether it induces apoptosis in the Caco-2 and C3A cell lines by means of flow cytometry and caspase-3 activation assay. We employed the Annexin-V-Fluorescein and Proidium iodid (PI) apoptosis assay, which is a popular method to distinguish between healthy cells (annexin V-negative; PI-negative), early apoptotic cells (annexin V-positive; PI-negative), late apoptotic cells (annexin V-positive; PI-positive), and necrotic cells (annexin V-negative; PI-positive). The C3A and Caco-2 cell lines were treated with different concentrations of compound **4g** (5 and 12.5 µM) for 24 h and then stained with propidium iodide (PI) and annexin V binding buffer. The percentage of cell populations, namely, necrotic cell (Q1), late apoptotic cells (Q2), healthy cells (Q3), and early apoptotic cells (Q4) are represented in Figure 2. Both necrotic (Q1) and apoptotic cells (Q2) increased with the increasing concentration, but the population of necrotic cells remained smaller at both concentrations tested. The population of necrotic (Q1) and apoptotic cells (Q2) in the case of the Caco-2 cells is almost equal at the highest concentration tested. Both necrotic and apoptotic cells in the case of C3A increased with increasing concentration of **4g**, but the population of necrotic cells remained higher. This is presumably because necrosis takes place after the cells have already died and reached equilibrium with their surroundings [31,32].

**Figure 3:**
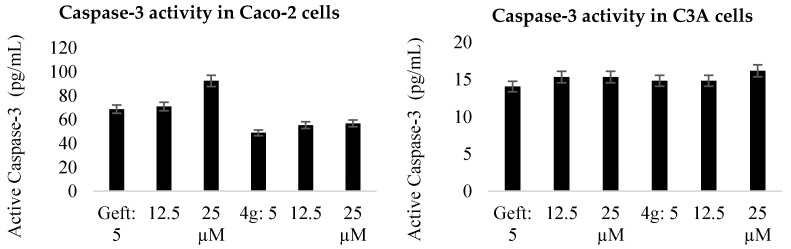
Effects of compounds EGFR on Caspace-3 activity in Caco-2 and C3A cells compared to gefitinib.

The molecular definition of apoptosis is based on the proteolytic activity of certain caspases (caspace-2, -3, -6, -7, -8, -9, and -10), and these enzymes are directly involved in the mediation of apoptotic cell death [32]. Activation of caspase-3, for example, is critically important for the induction of apoptotic pathways [33,34,35]. As a result, we evaluated compound EGFR for caspase-3 activation in Caco-2 and C3A cells against gefitinib as a reference standard (Figure 3). The observed results indicate that compound EGFR induces apoptosis in Caco-2 and C3A cells through activation of caspase that subsequently leads to cell membrane alterations observed using the staining technique above.

Gefitinib is a selective inhibitor of the EGFR-TK [3] and an increased expression of this receptor is the hallmark of ovarian cancer, breast cancer, squamous cell carcinoma of the head and neck, and prostate cancer [36]. Inhibition of EGFR-TK activity is regarded as the most promising approach for innovative therapeutic strategies in cancer treatment. Human lung cancer (A549), epithelial colorectal adenocarcinoma (Caco-2) and hepatocellular carcinoma (C3A) cells have been found to express high levels of EGFR and to be sensitive to gefitinib [25,37,38]. Breast adenocarcinoma (MCF-7) and cervical cancer (HeLa), on the other hand, have moderate [39] and low EGFR expression [40], respectively. In order to test whether the indole-aminoquinazolines prepared in this investigation could inhibit the ligand binding-induced receptor phosphorylation, we performed kinase activity of EGFR in the presence of representative compounds **4f** and **4g**.

#### 2.2.3. Evaluation of Compound **4g** for Potential to Inhibit EGFR-TK Phosphorylation

**Figure 4:**
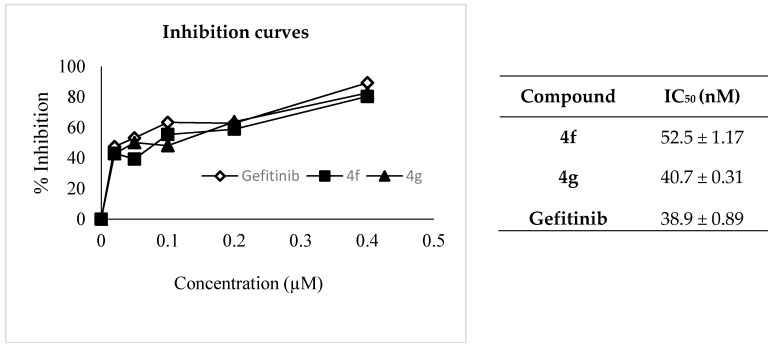
%Inhibition and IC_50_ values (in nM) of epidermal growth factor receptor tyrosine kinase (EGFR-TK) by **4f** and **4g** against gefitinib.

The inhibitory activities of compounds **4f** and **4g** to EGFR were tested by ELISA method against gefitinib as a reference standard. As shown in Figure 4, compounds **4f** and **4g** showed moderate (LC_50_ = 52.5 nM) and significant (LC_50_ = 40.7 nM) EGFR inhibitory activity, respectively, when compared to gefitinib (LC_50_ = 38.9 nM).

We rationalised the difference in cytotoxicity and EGFR-TK phosphorylation inhibitory activity related to the substitution pattern on the indole arm to be probably associated with the flexibility of the amino-indole moiety, where a distinct spatial arrangement of the molecule could lead to an improved interaction with the active site of the receptor. To try to prove this assumption, we performed molecular docking of compounds **4a**–**h** into EGFR-TK active site to compare the binding conformation with gefitinib and erlotinib.

#### 2.2.4. Molecular Docking Studies of Compounds **4a**–**h** into the ATP Binding Site of EGFR

A crystal structure of EGRF that previously co-crystalised with erlotinib was obtained from the protein data bank (PDB ID: 1M17). The control (gefitinib) docked on erlotinib binding site produced root mean square deviation (RMSD) of 1.6 Å with the crystal structure (Table 3). Comparison of the calculated binding free energy reveals that gefitinib is more favourable for the inhibition of EGFR compared with erlotinib. The gefitinib morpholine region was positioned as anti-parallel with the benzene ring of Phe699, but not for erlotinib. Besides, the 3-chloro-4-fluorophenyl moiety of gefitinib is docked deeper into the binding pocket compared to erlotinib. The N-morpholine region of gefitinib is also extended outward compared with erlotinib. The binding mode of gefitinib could be the reason for a better binding affinity compared with erlotinib and hence inherent selectivity for the EGFR. Compound **4e** showed the highest binding affinity among the hybrids with binding free energy of −11.46 kcal/mol.

**Figure 5:**
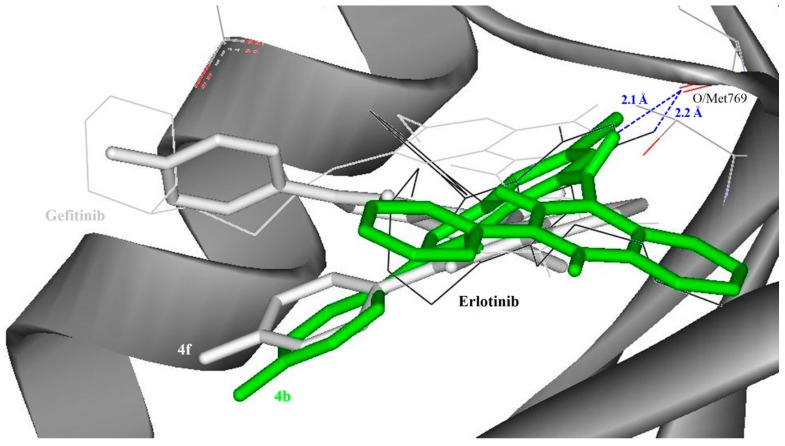
Docked conformation of erlotinib (as control), gefitinib, indole-aminoquinazolines **4b** and **4f** (stick representation) in the binding pocket of the EGFR kinase domain (surface and ribbon representation). Blue dotted lines indicate the direct hydrogen bonding formed between the docked ligand and EGFR. The docked conformation of other indole-aminoquinazolines can be found in Supplementary data Figure S2.

The binding modes of the indole-aminoquinazoline hybrids **4b** and **4f** are shown in Figure 5 (refer to Figure S2, Supplementary Data for the superimposed docking poses of **4a**–**h**). The compounds (**4b** and **4f**) bind on the same binding site with erlotinib than the EGFR selective gefitinib when compared with other compounds, i.e., **4a** and **4e**. The quinazoline region and the benzene were positioned same with the ether tails of erlotinib. Previous studies on the 4-anilinoquinazolines have shown that the 4-anilino group can fit deep into the hydrophobic pocket of EGFR [41], and that the introduction of electron-donating groups on the phenyl ring increase the electron density on the quinazoline N1 thereby enhancing the interaction with EGFR [42].

Compound **4b** substituted with a phenyl group on the quinazoline ring and a small lipophilic moderately pi-electron donating fluorine atom at the 4-position of the phenyl ring of the indole. This showed moderate to significant cytotoxicity against the five cell lines tested was docked deeper into the binding pocket compared with the other derivatives. Direct hydrogen bond formation was only observed between EGFR, with erlotinib and quinazoline-indole hybrids **4a**, **4b** and **4e**. The observed hydrogen bonds in the indole-aminoquinazoline hybrids demonstrate the importance of the amine moiety in this interaction. Compounds **4a** and **4e** adopted similar poses where the aryl substituent of the quinazoline moiety extended towards the entrance of the binding site and formed T-shape π-stacking interaction with Phe699. Compounds **4c**, **d**, and **f**–**h**, on the other hand, have similar binding conformation with their aryl/fluorophenyl group extended from quinazoline region forming parallel displaced π-stacking interactions with Phe699. The calculated binding free energies for gefitinib (−10.74 kcal/mol), **4f** (−9.67 kcal/mol), and **4g** (−10.30 kcal/mol) are consistent with their inhibitory effects against EGFR with IC_50_ values of 38.9, 52.5 and 40.7 nM, respectively. The calculated binding free energies show that the compounds have high affinity for the EGFR than erlotinib, but relatively less than that of most selective EGFR inhibitor, gefitinib. Besides, the calculated K*i* value of the control erlotinib that is 30 × higher than that of gefitinib is also evidence for the EGFR selectivity for gefitinib.

## 3. Materials and Methods

### 3.1. General

The melting points of the compounds prepared in this investigation were recorded on a Thermocouple digital melting point apparatus (Mettler Toledo LLC, Columbus, OH, USA). Their IR spectra were recorded as powders using a Bruker VERTEX 70 FT-IR Spectrometer (Bruker Optics, Billerica, MA, USA) equipped with a diamond attenuated total reflectance (ATR) accessory. For column chromatography, we used the Merck kieselgel 60 (0.063–0.200 mm) (Merck KGaA, Frankfurt, Germany) as stationary phase. The NMR spectra were obtained as DMSO-*d*_6_ solutions using Varian Mercury 300 MHz NMR spectrometer (Varian Inc., Palo Alto, CA, USA) and the chemical shifts are quoted relative to the residual proton signal in the deuterated solvent. The mass spectra were recorded at an ionization potential of 70 eV using Waters Synapt G2 Quadrupole Time-of-flight mass spectrometer (Waters Corp., Milford, MA, USA) at the University of Stellenbosch. The synthesis and analytical data of compounds **1a**–**d**, which are known have been described before [18].

### 3.2. Typical Procedure for the Preparation of the 2-Aryl-4-chloroquinazolines (***3a*** and ***3b***)

2-Arylquinazolin-4(3*H*)-one (2.00 g) was added in portions to a stirred SOCl_2_ (10 mL), followed by DMF (1 mL). the mixture was then refluxed for 2 h at 80 °C. The reaction mixture was cooled and then quenched slowly with saturated aqueous solution of K_2_CO_3_. The resultant precipitate was filtered and recrystallised from acetonitrile to give pure 2-aryl-4-chloroquinazoline as solid. Compounds **3a** and **3d** described below were prepared in this fashion.

#### 3.2.1. 4-Chloro-2-phenylquinazoline (**3a**)

Solid (1.90 g, 86%), mp. 124−126 °C (Lit. [21] 123−125 °C); ^1^H-NMR (DMSO-*d*_6_) 7.56 (4H, m, ArH), 7.90 (1H, t, *J* = 6.9 Hz, ArH), 8.04 (4H, m, ArH).

#### 3.2.2. 4-Chloro-2-(4-fluorophenyl)quinazoline (**3b**)

Solid (1.81 g, 84%), *R_f_* (60% EtOAc–hexane) = 0.81, mp. 139−141 °C; ν_max_ (ATR) 1602 (C=N), 1645 (C=N) cm^−1^; ^1^H-NMR (DMSO-*d*_6_) 7.36 (2H, t, *J* = 8.7 Hz, ArH), 7.49 (1H, t, *J* = 6.9 Hz, ArH), 7.71 (1H, d, *J* = 8.1 Hz, ArH), 7.80 (1H, t, *J* = 6.9 Hz, ArH), 8.11 (1H, d, *J* = 8.1 Hz, ArH), 8.19 (2H, dd, *J* = 5.7 and 8.7 Hz, ArH); ^13^C-NMR (DMSO-*d*_6_) 116.1 (d, ^2^*J*_CF_ = 21.8 Hz), 121.2, 126.3, 127.2, 127.3, 129.2 (d, ^4^*J*_CF_ = 2.3 Hz), 131.0 (d, ^3^*J*_CF_ = 9.1 Hz), 135.2, 148.3, 152.2, 162.5, 164.4 (d, ^1^*J*_CF_ = 248.4 Hz); HRMS (ES): Found: 259.0432. C_14_H_9_N_2_ClF^+^ requires 259.0438.

### 3.3. Typical Procedure for the Hydrolysis of the 7-Acetamide Derivatives ***1a***–***d***

A mixture of 1 (1 equivalent) and SOCl_2_ (4 equivalent) in methanol (15 mL/mmol of 1) was stirred under reflux for 1 h. The reaction mixture was cooled to room temperature (RT) and quenched with a saturated solution of K_2_CO_3_. The product was extracted with CHCl_3_ (3 × 20 mL), and then combined organic layers were dried over anhydrous MgSO_4_. The salt was filtered off and the solvent was evaporated under reduced pressure on a rotary evaporator. The residue was purified by column chromatography on silica gel using 60% EtOAc-hexane mixture as an eluent to afford 2 as a solid. The following compounds were prepared in this fashion.

#### 3.3.1. 5-Bromo-2-phenyl-1*H*-indol-7-amine (**2a**)

Solid (0.13 g, 78%), *R_f_* = 0.74, mp. 222‒223 °C; ν_max_ (ATR) 1578 (C=N), 3222 (NH), 3294 (NH), 3367 (NH) cm^−1^; ^1^H-NMR (DMSO-*d*_6_) 5.48 (2H, br s, NH_2_), 6.46 (1H, s, 3-H), 6.74 (1H, d, *J* = 1.2 Hz, 6-H), 6.91 (1H, d, *J* = 1.2 Hz, 4-H), 7.31 (1H, t, *J* = 7.5 Hz, 4′-H), 7.47 (2H, t, *J* = 7.5 Hz, 3′,5′-H), 7.80 (2H, d, *J* = 7.5 Hz, 2′,6′-H), 11.10 (1H, s, NH); ^13^C-NMR (DMSO-*d*_6_) 99.3, 107.6, 110.6, 113.6, 125.2, 125.3, 128.0, 129.4, 130.9, 132.4, 135.9, 137.8; HRMS (ES): Found 287.0187. C_14_H_12_N_2_^79^Br^+^ requires 287.0184. *Anal* calcd for C_14_H_11_N_2_Br: C, 58.56; H, 3.86; N, 9.76. Found: C, 58.49; H, 3.82; N, 9.76.

#### 3.3.2. 5-Bromo-2-(4-fluorophenyl)-1*H*-indol-7-amine (**2b**)

Solid (0.14 g, 80%), *R_f_* = 0.76, mp. 260‒263 °C; ν_max_ (ATR) 1570 (C=N), 3209 (NH), 3296 (NH), 3368 (NH) cm^−1^; ^1^H-NMR (DMSO-*d*_6_) 6.35 (2H, br s, NH_2_), 6.60 (1H, s, 3-H), 6.76 (1H, d, *J* = 1.2 Hz, 6-H), 7.04 (1H, d, *J* = 1.2 Hz, 4-H), 7.34 (2H, t, *J* = 8.7 Hz, 3′,5′-H), 7.89 (2H, d, *J* = 8.7 Hz, 2′,6′-H), 11.40 (1H, s, NH); ^13^C NMR (DMSO-*d*_6_) 99.2, 109.2, 112.2, 113.3, 116.4 (d, ^2^*J*_CF_ = 21.8 Hz), 126.0, 127.4 (d, ^3^*J*_CF_ = 8.0 Hz), 128.9 (d, ^4^*J*_CF_ = 2.3 Hz), 131.1, 133.3, 137.3, 162.1 (d, ^1^*J*_CF_ = 243.9 Hz); HRMS (ES): Found 305.0082. C_14_H_11_N_2_F^79^Br^+^ requires 305.0090. *Anal* calcd for C_14_H_10_N_2_FBr: C, 55.10; H, 3.30; N, 9.18. Found: C, 54.97; H, 3.13; N, 8.89.

#### 3.3.3. 5-Bromo-2-(3-chlorophenyl)-1*H*-indol-7-amine (**2c**)

Solid (0.13 g, 76%), *R_f_* = 0.82, mp. 271‒274 °C; ν_max_ (ATR) 1572 (C=N), 3213 (NH), 3289 (NH), 3356 (NH) cm^−1^; ^1^H-NMR (DMSO-*d*_6_) 6.36 (2H, br s, NH_2_), 6.77 (1H, s, 3-H), 6.96 (1H, d, *J* = 1.2 Hz, 6-H), 7.24 (1H, d, *J* = 1.2 Hz, 4-H), 7.38‒7.41 (1H, m, 4′-H), 7.51 (1H, t, *J* = 8.4 Hz, 5′-H), 7.86 (1H, d, *J* = 8.4 Hz, 6′-H), 7.96 (1H, s, 2′-H), 11.79 (1H, s, NH); ^13^C-NMR (DMSO-*d*_6_) 100.5, 112.1, 112.9, 115.1, 124.2, 124.9, 127.2, 127.9, 129.4, 131.3, 131.4, 134.1, 134.3, 137.2; HRMS (ES): Found 320.9778. C_14_H_11_N_2_^35^Cl^79^Br^+^ requires 320.9794. *Anal* calcd for C_14_H_10_N_2_ClBr: C, 52.29; H, 3.13; N, 8.71. Found: C, 52.17; H, 2.98; N, 8.56.

#### 3.3.4. 5-Bromo-2-(4-methoxyphenyl)-1*H*-indol-7-amine (**2d**)

Solid (0.13 g, 76%), *R_f_* = 0.61, mp. 223‒224 °C; ν_max_ (ATR) 1550 (C=N), 1609 (C=N), 3220 (NH), 3232 (NH), 3367 (NH) cm^−1^; ^1^H-NMR (DMSO-*d*_6_) 3.80 (3H, s, OCH_3_), 5.42 (2H, br s, NH_2_), 6.43 (1H, s, 3-H), 6.60 (1H, d, *J* = 1.2 Hz, 6-H), 6.87 (1H, d, *J* = 1.2 Hz, 4-H), 7.04 (2H, d, *J* = 8.7 Hz, 3′,5′-H), 7.72 (2H, d, *J* = 8.7 Hz, 2′,6′-H), 10.97 (1H, s, NH); ^13^C-NMR (DMSO-*d*_6_) 55.7, 98.0, 107.3, 110.3, 113.4, 114.9, 125.0, 125.1, 126.7, 131.1, 135.7, 138.0, 159.3; HRMS (ES): Found: 317.0278. C_15_H_14_N_2_^79^BrO^+^ requires 317.0290. *Anal* calcd for C_15_H_13_N_2_FBrO: C, 56.80; H, 4.13; N, 8.83. Found: C, 56.74; H, 4.01; N, 8.86.

### 3.4. Typical Procedure for the Amination of the Synthesis of ***4a***–***h***

A mixture of **2** (1 equiv.), 2-aryl-4-chloroquinazoline (1 equiv.) and HCl (5% mol equiv. of 2) in 3:1 isopropanol-THF (*v*/*v*, 30 mL/mmol of 2) was stirred under reflux for 2 h. After completion of the reaction (tlc monitoring) the solvent was evaporated under reduced pressure and the residue obtained was recrystallised from chloroform to obtain pure 4. The following compounds were prepared in this fashion:

#### 3.4.1. *N*-(5-Bromo-2-phenyl-1*H*-indol-7-yl)-2-phenylquinazolin-4-amine (**4a**)

Solid (0.21 g, 81%), mp. > 300 °C; ν_max_ (ATR) 1540 (C=N), 1604 (C=N), 3052 (NH), 3197 (NH) cm^−1^; ^1^H-NMR (DMSO-*d*_6_) 6.99 (1H, s, Ar), 7.25‒7.37 (5H, m, Ar), 7.50‒7.54 (2H, m, Ar), 7.74‒7.79 (3H, m, Ar), 7.86 (1H, t, *J* = 7.5 Hz, Ar), 8.07‒8.14 (3H, m, Ar), 8.48 (1H, d, *J* = 8.1 Hz, Ar), 9.06 (1H, d, *J* = 8.1 Hz, Ar), 11.42 (1H, s, NH), 12.16 (1H, br s, NH); ^13^C-NMR (DMSO-*d*_6_) 99.5, 111.2, 113.8, 121.2, 121.6, 123.1, 125.5, 126.2 (2C), 128.3, 128.5, 129.1 (2C), 129.2 (2C), 129.4, 131.2, 131.8, 132.3, 133.5, 136.0, 140.4, 157.6, 160.0; HRMS (ES): Found 491.0836. C_28_H_20_N_4_^79^Br^+^ requires 491.0871. *Anal* calcd for C_28_H_19_N_4_Br: C, 68.44; H, 3.90; N, 11.40. Found: C, 68.43; H, 3.75; N, 11.23.

#### 3.4.2. *N*-(5-Bromo-2-(4-fluorophenyl)-1*H*-indol-7-yl)-2-phenylquinazolin-4-amine (**4b**)

Solid (0.19 g, 79%), mp. > 300 °C; ν_max_ (ATR) 1557 (C=N), 1601 (C=N), 3052 (NH), 3198 (NH) cm^−1^; ^1^H-NMR (DMSO-*d*_6_) 6.96 (1H, s, Ar), 7.18 (2H, t, *J* = 7.5 Hz, Ar), 7.25‒7.34 (2H, m, Ar), 7.51 (2H, d, *J* = 1.5 Hz, Ar), 7.76‒7.80 (3H, m, Ar), 7.88 (1H, t, *J* = 7.5 Hz, Ar), 8.07‒8.09 (3H, m, Ar), 8.49 (1H, d, *J* = 8.1 Hz, Ar), 9.10 (1H, d, *J* = 8.1 Hz, Ar), 11.44 (1H, s, NH), 12.19 (1H, br s, NH); ^13^C-NMR (DMSO-*d*_6_) 99.5, 111.2, 113.6, 116.2 (d, ^2^*J*_CF_ = 21.8 Hz), 120.8 (d, ^4^*J*_CF_ = 3.5 Hz), 121.3, 121.9, 122.8, 125.5, 128.3 (d, ^3^*J*_CF_ = 8.0 Hz), 128.6, 129.2 (2C), 129.4, 131.2, 131.6, 132.3, 133.8, 136.4, 139.4, 140.8, 157.5, 160.1, 162.3 (d, ^1^*J*_CF_ = 243.9 Hz); HRMS (ES): Found 509.0757. C_28_H_19_N_4_F^79^Br^+^ requires 509.0777. *Anal* calcd for C_28_H_18_N_4_FBr: C, 66.02; H, 3.56; N, 11.00. Found: C, 66.11; H, 3.52; N, 10.79.

#### 3.4.3. *N*-(5-Bromo-2-(3-chlorophenyl)-1*H*-indol-7-yl)-2-phenylquinazolin-4-amine (**4c**)

Solid (0.18 g, 75%), mp. 228‒230 °C; ν_max_ (ATR) 1545 (C=N), 1600 (C=N), 3060 (NH), 3186 (NH) cm^−1^; ^1^H-NMR (DMSO-*d*_6_) 7.11 (1H, s, Ar), 7.30‒7.41 (4H, m, 4H), 7.52‒7.57 (2H, m, Ar), 7.74‒7.77 (1H, m, Ar), 7.80 (1H, t, *J* = 1.8 Hz, Ar), 7.82 (1H, d, *J* = 1.5, Ar), 7.88 (1H, t, *J* = 7.5 Hz, Ar), 8.05‒8.08 (2H, m, Ar), 8.14 (1H, t, *J* = 7.5 Hz, Ar), 8.45 (1H, d, *J* = 8.1 Hz, Ar), 9.05 (1H, d, *J* = 8.1 Hz, Ar), 11.46 (1H, s, NH), 12.14 (1H, br s, NH); ^13^C-NMR (DMSO-*d*_6_) 100.2, 111.3, 113.6, 120.7, 121.8, 122.2, 123.0, 124.9, 125.6, 128.2, 128.3, 128.6, 129.1, 129.2, 129.4, 131.0, 131.4, 131.5, 132.1, 133.8, 134.1, 136.4, 138.7, 140.7, 157.5, 160.1; HRMS (ES): Found 525.0495. C_28_H_19_N_4_^35^Cl^79^Br^+^ requires 525.0482. *Anal* calcd for C_28_H_18_N_4_ClBr: C, 63.96; H, 3.45; N, 10.66. Found: C, 63.93; H, 3.46; N, 10.59.

#### 3.4.4. *N*-(5-Bromo-2-(4-methoxyphenyl)-1*H*-indol-7-yl)-2-phenylquinazolin-4-amine (**4d**)

Solid (0.21 g, 83%), mp. 261‒264 °C; ν_max_ (ATR) 1547 (C=N), 1609 (C=N), 3060 (NH), 3134 (NH) cm^−1^; ^1^H-NMR (DMSO-*d*_6_) 3.72 (3H, s, CH_3_), 6.88 (1H, s, Ar), 6.92 (2H, d, *J* = 8.7 Hz, Ar), 7.36 (2H, t, *J* = 7.5 Hz, Ar), 7.47 (1H, d, *J* = 1.5 Hz, Ar), 7.52‒7.57 (1H, m, Ar), 7.70 (2H, d, *J* = 8.7 Hz, Ar), 7.76 (1H, d, *J* = 1.5 Hz, Ar), 7.88 (1H, t, *J* = 7.5 Hz, Ar), 8.08‒8.16 (3H, m, Ar), 8.53 (1H, d, *J* = 8.1 Hz, Ar), 9.09 (1H, d, *J* = 8.1 Hz, Ar), 11.32 (1H, s, NH), 12.21 (1H, br s, NH); ^13^C-NMR (DMSO-*d*_6_) 54.2, 96.9, 109.6, 112.3, 113,2 119.1, 119.5, 120.2, 121.1, 122.9, 124.2, 126.3, 127.2, 127.8, 128.2, 129.7, 130.0, 131.2, 132.6, 135.0, 139.1, 139.2, 155.0, 158.3, 158.7; HRMS (ES): Found 521.0987. C_29_H_22_N_4_^79^BrO^+^ requires 521.0977. *Anal* calcd for C_29_H_21_N_4_BrO: C, 66.80; H, 4.06; N, 3.07. Found: C, 66.87; H, 3.99; N, 3.01.

#### 3.4.5. *N*-(5-Bromo-2-phenyl-1*H*-indol-7-yl)-2-(4-fluorophenyl)quinazolin-4-amine (**4e**)

Solid (0.21 g, 78%), mp. > 300 °C; ν_max_ (ATR) 1545 (C=N), 1603 (C=N), 3065 (NH), 3199 (NH) cm^−1^; ^1^H-NMR (DMSO-*d*_6_) 6.99 (1H, s, Ar), 7.18‒7.28 (3H, m, Ar), 7.34 (2H, t, *J* = 7.2 Hz, Ar), 7.51 (1H, d, *J* = 1.5 Hz, Ar), 7.72‒7.88 (4H, m, Ar), 8.09‒8.21 (3H, m, Ar), 8.55 (1H, d, *J* = 8.1 Hz, Ar), 9.11 (1H, d, *J* = 8.1 Hz, Ar), 11.40 (1H, s, NH), 12.30 (1H, br s, NH); ^13^C-NMR (DMSO-*d*_6_) 99.6, 111.1, 113.7, 116.3 (d, ^2^*J*_CF_ = 21.8 Hz), 121.3, 121.8, 122.9, 125.5, 126.2 (2 × C), 128.4 (d, ^3^*J*_CF_ = 8.0 Hz), 128.5, 129.2 (2C), 131.1, 131.8, 132.1, 132.2 (d, ^4^*J*_CF_ = 5.7 Hz), 132.3, 136.2, 140.4, 156.5, 159.9, 165.4 (d, ^1^*J*_CF_ = 250.7 Hz; HRMS (ES): Found 509.0769. C_28_H_19_N_4_F^79^Br^+^ requires 509.0777. *Anal* calcd for C_28_H_18_N_4_FBr: C, 66.02; H, 3.56; N, 11.00. Found: C, 59.97; H, 3.46; N, 10.78.

#### 3.4.6. *N*-(5-Bromo-2-(4-fluorophenyl)-1*H*-indol-7-yl)-2-(4-fluorophenyl)quinazolin-4-amine (**4f**)

Solid (0.20 g, 80%), mp. > 300 °C; ν_max_ (ATR) 1557 (C=N), 1605 (C=N), 3039 (NH), 3259 (NH) cm^−1^; ^1^H-NMR (DMSO-*d*_6_) 6.97 (1H, s, Ar), 7.21 (2H, t, *J* = 8.7 Hz, Ar), 7.24 (2H, t, *J* = 8.7 Hz, Ar), 7.49 (1H, d, *J* = 1.5 Hz, Ar), 7.80‒7.90 (4H, m, Ar), 8.10‒8.15 (3H, m, Ar), 8.47 (1H, d, *J* = 8.1 Hz, Ar), 9.03 (1H, d, *J* = 8.1 Hz, Ar), 11.40 (1H, s, NH), 12.13 (1H, br s, NH); ^13^C-NMR (DMSO-*d*_6_) 99.6, 111.2 (2C), 116.2 (d, ^2^*J*_CF_ = 21.8 Hz), 116.4 (d, ^2^*J*_CF_ = 22.9 Hz), 121.0 (d, ^4^*J*_CF_ = 3.5 Hz), 121.3, 121.9, 122.8, 125.5, 128.3 (d, ^3^*J*_CF_ = 9.1 Hz), 128.4 (2C), 128.6, 131.2, 132.2 (d, ^3^*J*_CF_ = 9.1 Hz), 132.3 (d, ^4^*J*_CF_ 4.5 Hz), 136.3, 139.4 (2C), 156.5, 160.0, 162.3 (d, ^1^*J*_CF_ = 243.9 Hz), 165.5 (d, ^1^*J*_CF_ = 249.6 Hz); HRMS (ES): Found 527.0689. C_28_H_18_N_4_F_2_^79^Br^+^ requires 527.0683. *Anal* calcd for C_28_H_17_N_4_FBr: C, 63.77; H, 3.25; N, 10.62. Found: C, 63.80; H, 3.22; N, 10.53.

#### 3.4.7. *N*-(5-Bromo-2-(4-chlorophenyl)-1*H*-indol-7-yl)-2-(4-fluorophenyl)quinazolin-4-amine (**4g**)

Solid (0.20 g, 78%), mp. 266‒269 °C; ν_max_ (ATR) 1557 (C=N), 1605 (C=N), 3038 (NH), 3136 (NH) cm^−1^; ^1^H-NMR (DMSO-*d*_6_) 7.09 (1H, s, Ar), 7.28 9 (2H, t, *J* = 8.7 Hz, Ar), 7.30‒7.41 (2H, m, Ar), 7.54 (1H, d, *J* = 1.5 Hz, Ar), 7.72‒7.77 (2H, m, Ar), 7.82 (1H, d, *J* = 1.5 Hz, Ar), 7.87 (1H, t, *J* = 7.8 Hz, Ar), 8.10‒8.20 (3H, m, Ar), 8.47 (1H, d, *J* = 8.1 Hz, Ar), 9.05 (1H, d, *J* = 8.1 Hz, Ar), 11.44 (1H, s, NH), 12.23 (1H, br s, NH); ^13^C-NMR (DMSO-*d*_6_) 100.8, 111.3, 113.6, 116.4 (d, ^2^*J*_CF_ = 21.8 Hz), 121.8, 122.1, 123.1, 124.9, 125.5, 125.6, 127.1, 128.0, 128.2, 128.5, 131.0, 131.1, 131.3, 132.0, 132.1 (d, ^3^*J*_CF_ = 8.0 Hz), 133.8, 134.1, 136.3 (d, ^4^*J*_CF_ = 3.4 Hz), 138.6, 156.6, 160.0, 165.5 (d, ^1^*J*_CF_ = 250.7 Hz); HRMS (ES): Found 543.0377. C_28_H_18_N_4_F^35^Cl^79^Br ^+^ requires 543.0387. *Anal* calcd for C_28_H_17_N_4_FClBr: C, 61.84; H, 3.15; N, 10.30. Found: C, 61.79; H, 3.00; N, 10.12.

#### 3.4.8. *N*-(5-Bromo-2-(4-methoxyphenyl)-1*H*-indol-7-yl)-2-(4-fluorophenyl)quinazolin-4-amine (**4h**)

Solid (0.17 g, 67%), mp. 250‒253 °C; ν_max_ (ATR) 1553 (C=N), 1607 (C=N), 3037 (NH), 3187 (NH) cm^−1^; ^1^H-NMR (DMSO-*d*_6_) 3.68 (3H, s, CH_3_), 6.82 (1H, s, Ar), 6.88 (2H, d, *J* = 8.7 Hz, Ar), 7.20 (2H, t, *J* = 8.7 Hz, Ar), 7.41 (1H, d, *J* = 1.5 Hz, Ar), 7.64 (2H, d, *J* = 8.1 Hz, Ar), 7.71 (1H, d, *J* = 1.5 Hz, Ar), 7.82 (1H, t, *J* = 7.8 Hz, Ar), 8.05−8.14 (3H, m, Ar), 8.40 (1H, d, *J* = 8.1 Hz, Ar), 8.95 (1H, d, *J* = 8.1 Hz, Ar), 11.24 (1H, s, NH), 11.97 (1H, br s, NH); ^13^C-NMR (DMSO-*d*_6_) 55.6, 98.3, 111.0, 113.6, 114.6 (2C), 116.3 (d, ^2^*J*_CF_ = 21.8 Hz), 120.7, 121.3, 122.0, 124.3, 125.3, 127.7 (2C), 128.4, 131.0, 132.0 (d, ^3^*J*_CF_ = 9.1 Hz), 132.5, 136.2 6 (d, ^4^*J*_CF_ = 3.5 Hz), 140.5 (2C), 156.6, 159.7, 159.9, 165.4 (d, ^1^*J*_CF_ = 250.7 Hz); HRMS (ES): Found 539.0878. C_29_H_21_N_4_F^79^BrO^+^ requires 539.0883. *Anal* calcd for C_29_H_20_N_4_FBrO: C, 64.57; H, 3.74; N, 10.39. Found: C, 64.53; H, 3.70; N, 10.41.

### 3.5. Materials and Methods for In Vitro Cytotoxicity Assay of ***4a***–***h***

The cytotoxicity of the compounds were screened against four different cell lines: C3A/HepG2 human liver cells (derivatives of Hep G2 (ATCC HB8065)) (ATCC^®^ CRL10741), Caco2 (Human colorectal tumor cell), A549 (Human lung carcinoma), MCF-7 (breast adenocarcinoma), and HeLa (cervical cancer) cells. The lethal concentration was determined using the 3-(4,5-dimethylthiazol)-2,5-diphenyl tetrazolium bromide (MTT) assay [43]. The cells were maintained in Dubellsco minimal essential medium (DMEM, Highveld Biological, Sandton, South Africa) supplemented with 10% foetal calf serum (Adcock-Ingram, Midrand, South Africa) and sodium pyruvate for C3A liver cells, while Vero cells were supplemented with 5% foetal calf serum and 0.1% gentamicin (Virbac, Centurion, South Africa). Cell suspensions were prepared from confluent monolayer cultures and plated at a density of 0.1 × 10^6^ cells into each well of 96-well microtitre plates. For cell attachment, plates were incubated for 24 h at 37 °C in a 5% CO_2_ incubator prior to the exposure. The compounds were dissolved in DMSO (5 mg/mL), and appropriate dilutions were prepared, then added to the wells and incubated for 48h. Doxorubicin (Pfizer Laboratories, Sandton, South Africa) was used as a positive control while acetone was the negative control. After incubation for 48 h, the wells were rinsed with 150 µL of phosphate buffered saline PBS (Sigma-Aldrich, GmBH, Schnelldorf, Germany) and 200 µL of fresh medium was dispensed into the wells. MTT (Sigma) dissolved in PBS (30 µL) was added to each well and incubated for 4 h at 37 °C. The medium was removed and MTT formazan crystals were dissolved in 50 µL DMSO. Absorbance was measured on a BioTek microplate reader (BioTek Synergy, Analytical and Diagnostic Products, Johannesburg, South Africa) at a wavelength of 570 nm. Each concentration was tested in quadruplicate and the assay was repeated three times. The percentage of cell viability and the LC_50_ values for each compound tested were calculated as described before [15,17].

### 3.6. Apoptosis Assay

#### 3.6.1. Annexin V-FLUOS Staining Assay on **4g** against Caco-2 and C3A Cells

Apoptotic cells were quantified using flow cytometry. The Caco-2 or C3A cells were cultured in 6 well plates and each was then treated with compound **4g** at concentrations of 5, 12.5, and 25 µM, against doxorubicin hydrochloride (0.20 µM) as a reference standard. After the cells were incubated for 24 h, both treated and untreated cells were harvested, washed two times with ice PBS and then adjusted at a density of 1 × 10^6^ cells/sample. The cells were, in turn, stained with Annexin-V-FLUOS staining kit (Roche, Mannheim, Germany) according to the manufacturer’s instructions. The cells were analysed using Becton, Dicknson and Company FACS Accuri Flow Cytometer (Woodmead, South Africa).

#### 3.6.2. Caspace-3 Analysis on **4g** against C3A and Caco-2 Cells

Caspace-3 activity was detected by means of Caspace-3 Colorimetric Assay Kit (Abcam; Cambridge, MA, USA). The cells were cultured in 96 well plates and treated for 24 h. The cells were then washed with PBS buffer and lysed on ice the experiments were carried out according to the manufacturer’s instructions. Optical density was measured at absorbance of 405 nm using the BioTek microplate reader. The concentration of active Caspace-3 (Asp 175) were measured in duplicates and interpolated from the active caspase-3 (Asp 175) standard curve and corrected for sample dilution.

### 3.7. EGFR-TK Inhibition Assays

The inhibitory activities of compounds **4f**, **4g** and gefitinib towards EGFR-TK were tested using enzyme-linked immunosorbent assay (ELISA) technique with purified Epidermal Growth Factor Receptor (Sigma-Aldrich, Bradford, UK), following a procedure described in our previous investigation [17].

### 3.8. Molecular Docking of Compounds ***4a***–***h*** into EGFR-TK Active Site

The starting structure of EGFR kinase domain was retrieved from Research Collaboratory for Structural Bioinformatics Protein Data Bank (RCSB PDB) with the identity 1M17 [44]. All heteroatoms and water molecules were first removed. Polar hydrogen atoms, Kollman-Amber united atom partial charges and solvation parameters were then added by utilising AutoDockTools (The Scripps Research Institute, La Jolla, California, USA) [45]. The coordinates for control docking (erlotinib) was retrieved from the ligand of EGRF (PDB id: 1M17), while the coordinates for compounds **4a**–**h** were generated using ChemDraw Professional 15.0 (PerkinElmer Informatics, Waltham, MA, USA) and minimised with Chem3D module in ChemOffice Professional 15.0 (PerkinElmer Informatics). Only polar hydrogen remained. AutoDockTools was then used to assign Gasteiger charges and torsional angles to all ligands. Hydrated docking was applied in this work with standard protocol from AutoDock4.2 [45] where water atoms were added to the ligand. Docking was performed by AutoDock4.2.6 [45] with Lamarckian genetic algorithm of 1,000,000 energy evaluations per run and a maximum number of 27,000 generation. The corresponding docked conformations were clustered with root mean square of 2.0 Å.

## 4. Conclusions

We have demonstrated that compound **4g**, which exhibited significant cytotoxicity against several cancer cell lines tested was able to trigger apoptosis in Caco-2 and C3A cells. This demonstrates that the indole-aminoquinazoline hybrids prepared in this investigation possess in vitro antiproliferative and pro-apoptotic activity. Moreover, compound **4g** not only exhibited significant cytotoxicity against most of the cancer cell lines, but also showed significant inhibitory activity (IC_50_ = 40.7 nM) towards EGFR comparable to that of the medicinally important EGFR inhibitor, gefitinib (IC_50_ = 38.9 nM). It has been reported in the literature that bioisosteric replacement of hydrogen by fluorine in position 4 of the phenyl ring makes electronic modulation to reinforce and enhance the binding interaction process [46]. This probably accounts for the observed increased cytotoxicity of 4-fluorophenyl substituted derivatives, compounds **4b**, **4f** and **4g**, as well as their inhibitory effect against EGFR-TK phosphorylation. We attribute the difference in activity related to the halogen substitution pattern on the aminoindole arm to be associated with the flexibility of this moiety, where a distinct spatial arrangement of the molecule leads to an improved interaction with the active site of the receptor. Molecular docking (in silico) studies of compounds **4a**–**h** confirm the presence of hydrogen bonding between the indole–NH and the peptide backbone of the EGFR, which would account for the improved inhibitory activity of these compounds. Besides, the π-stacking interactions of the indole-aminoquinazoline hybrids with Phe699 could contribute to the favourable binding affinity with EGFR. The docking studies showed that these title compounds bind more like erlotinib than gefitinib, which is a selective inhibitor for the EGFR. This thus suggests that the title compounds may also target proteins other than EGFR. This in our view forms a basis for extension of this study to other types of protein kinases to explore the mechanism of their action and selectivity for specific type of protein kinase. These preliminary in vitro cytotoxicity results and SAR analysis form a basis for the design and synthesis of derivatives substituted on the 4-position of the phenyl ring with other lipophilic atoms or groups, such as the chlorine atom or a methyl group. This establishes the effect of lipophilicity of the substituents on the biological activity of these indole-aminoquinazoline hybrids.

## Figures and Tables

**Table 1:** Designation of R^1^ and R^2^ for products **4a**–**h**.

Compound	R^1^	R^2^
**4a**	C_6_H_5_-	C_6_H_5_-
**4b**	4-FC_6_H_4_-	C_6_H_5_-
**4c**	3-ClC_6_H_4_-	C_6_H_5_-
**4d**	4-CH_3_OC_6_H_4_-	C_6_H_5_-
**4e**	C_6_H_5_-	4-FC_6_H_4_-
**4f**	4-FC_6_H_4_-	4-FC_6_H_4_-
**4g**	3-ClC_6_H_4_-	4-FC_6_H_4_-
**4h**	4-CH_3_OC_6_H_4_-	4-FC_6_H_4_-

**Table 2:**
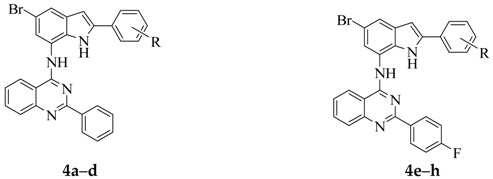
Cytotoxic effects of **4a**–**h** against the A549, Caco-2, C3A, MCF-7, and HeLa cell lines.

Ligand	Cancer Cells, LC_50_ (µM)				
	A549	Caco-2	C3A	MCF-7	HeLa
**4a**	60.99 ± 0.14	41.55 ± 0.01	30.41 ± 0.02	45.99 ± 0.08	32.23 ± 1.23
**4b**	32.04 ± 0.17	12.67 ± 0.00	17.65 ± 0.1	30.11 ± 1.21	24.70 ± 1.31
**4c**	36.97 ± 0.31	27.32 ± 0.01	30.57 ± 0.02	41.22 ± 0.09	40.02 ± 3.23
**4d**	>100	52.65 ± 0.00	59.97 ± 0.05	69.11 ± 0.12	61.23 ± 6.37
**4e**	51.64 ± 0.16	37.77 ± 0.10	38.75 ± 0.05	56.67 ± 0.45	43.37 ± 2.05
**4f**	17.66 ± 0.08	9.16 ± 0.01	19.92 ± 0.10	26.96 ± 2.30	34.05 ± 1.28
**4g**	86.21 ±0.14	6.45 ± 0.01	12.20 ± 0.03	39.07 ± 1.56	25.51 ± 2.94
**4h**	57.51 ± 0.21	38.76 ± 0.00	32.77 ± 0.07	61.03 ± 0.39	49.13 ± 1.10
**Gefitinib**	51.29 ± 0.17	27.91 ± 0.00	5.01 ± 0.04	30.74 ± 0.80	98.80 ± 0.56

**Table 3:** Calculated binding free energy and estimated inhibition constant (K*_i_*) of erlotinib (as control), gefitinib and quinazoline-indole hybrids **4a**–**h** with epidermal growth factor receptor (EGFR) kinase domain.

Ligand	Hb Distance (Å)	Hb Interacting Atoms	FEB (kcal/mol)	Ic, K*_i_* (nM)
Control (erlotinib)	2.2	Met769 O–Erl H_N2_	−8.71	414.12
Gefitinib	-	-	−10.74	13.34
**4a**	2.9 2.0	Thr766 O_G1_–4a H_N2_ Asp831 O_D1_–4a H_N_	−11.03	8.22
**4b**	2.1	Met769 O–4b H_N_	−9.95	50.7
**4c**	-	-	−10.10	39.74
**4d**	-	-	−10.31	27.74
**4e**	3.0	Asp831 O_D1_–4e H_N_	−11.46	4.01
**4f**	-	-	−9.67	81.76
**4g**	-	-	−10.30	28.38
**4h**	-	-	−10.36	25.61

Hb: Hydrogen bond; FEB: Free energy of binding; Ic: Estimated inhibition constant.

## Supplementary Materials

Supplementary materials can be found at http://www.mdpi.com/1422-0067/19/8/2232/s1.
