# Supplementary material for: Synthesis, Biological Evaluation and Molecular Docking of Novel Indole-Aminoquinazoline Hybrids for Anticancer Properties
Source: Int J Mol Sci. 2018 Jul 31;19(8):2232. doi: 10.3390/ijms19082232 (PMC6121530; doi:10.3390/ijms19082232)
Supplement: Supplementary file 1 [file ijms-19-02232-s001.zip › ijms-330585-SI.pdf]

Supplementary Information:

Synthesis, biological evaluation and molecular docking of novel indole-aminoquinazoline hybrids for anticancer properties

M.J. Mphahlele, M.M. Mmonwa, A. Aro, L.J. McGaw and Y.S. Choong

Figure S1: ^{1}H-NMR and ^{13}C-NMR spectra of compounds 2a–d and 4a–h

Figure S2: Docked conformation of erlotinib, gefitinib, indole-aminoquinazolines 4a–h

Int. J. Mol. Sci. 2018, 19, 2232; doi:10.3390/ijms19082232 www.mdpi.com/journal/ijms

Figure S1: ^{1}H-NMR and ^{13}C-NMR spectra of compounds 2a–d and 4a–h

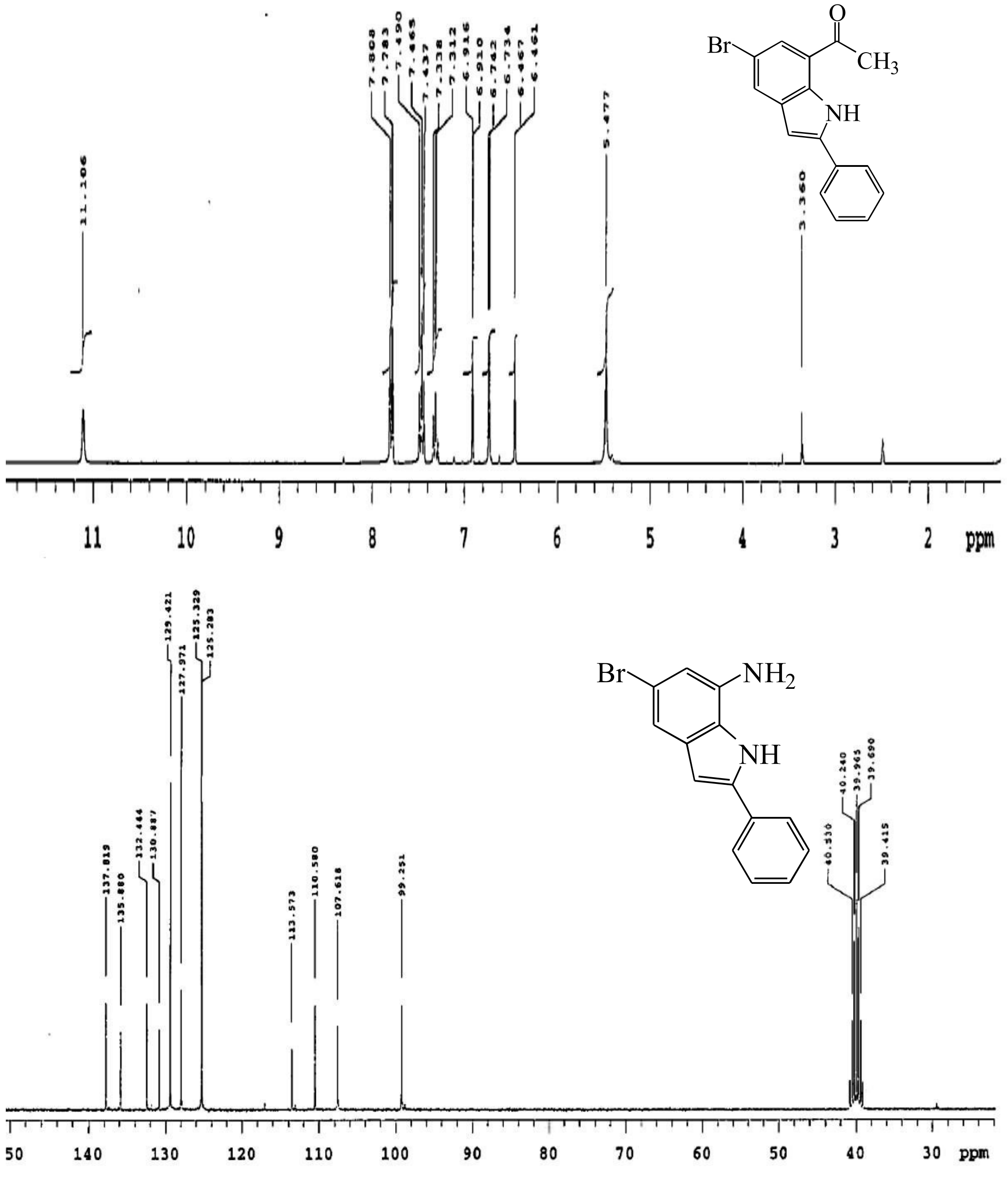

Figure S1.1: ^{1}H- and ^{13}C-NMR spectra of **2a** in DMSO-d_6 at 300 MHz and 75 MHz, respectively

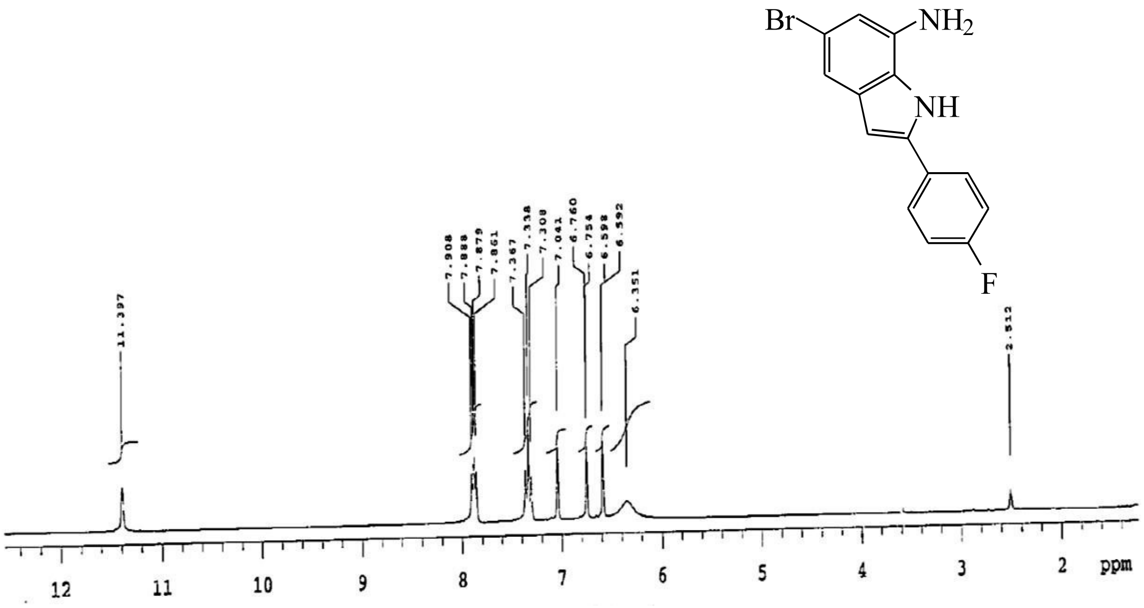

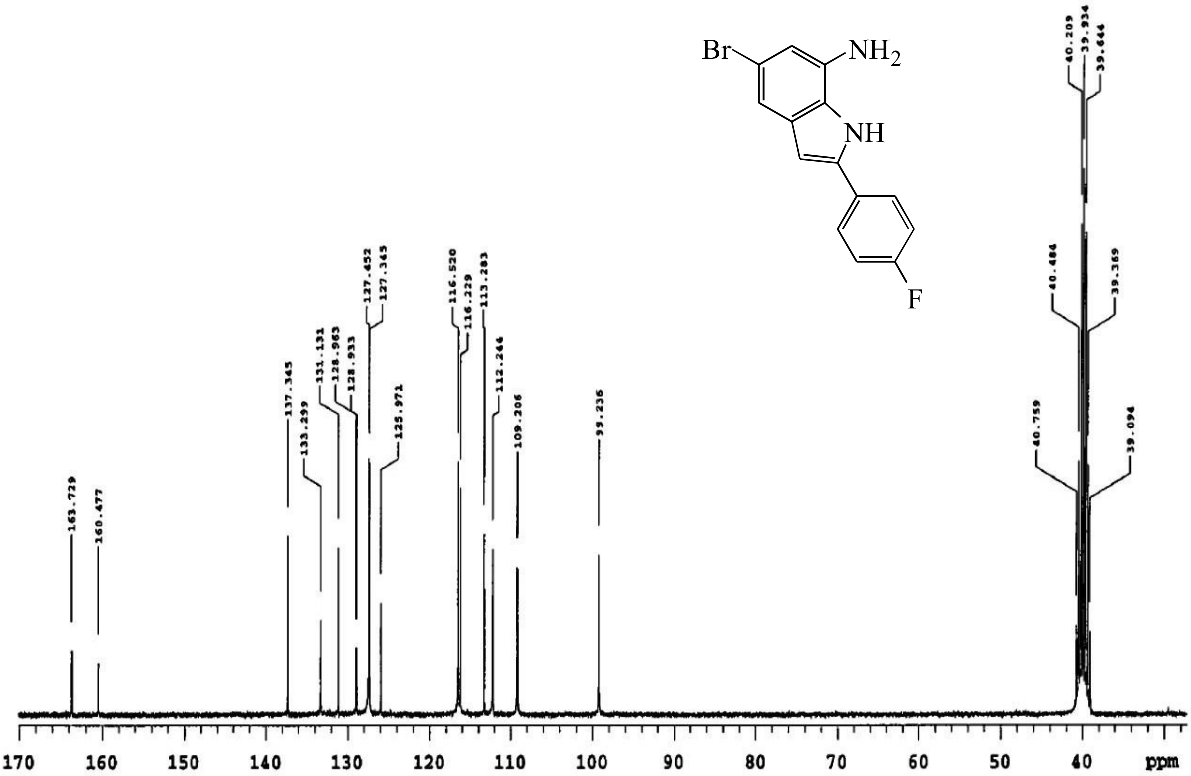

Figure S1.2: ^{1}H- and ^{13}C-NMR spectra of **2b** in DMSO-d_6 at 300 MHz and 75 MHz, respectively

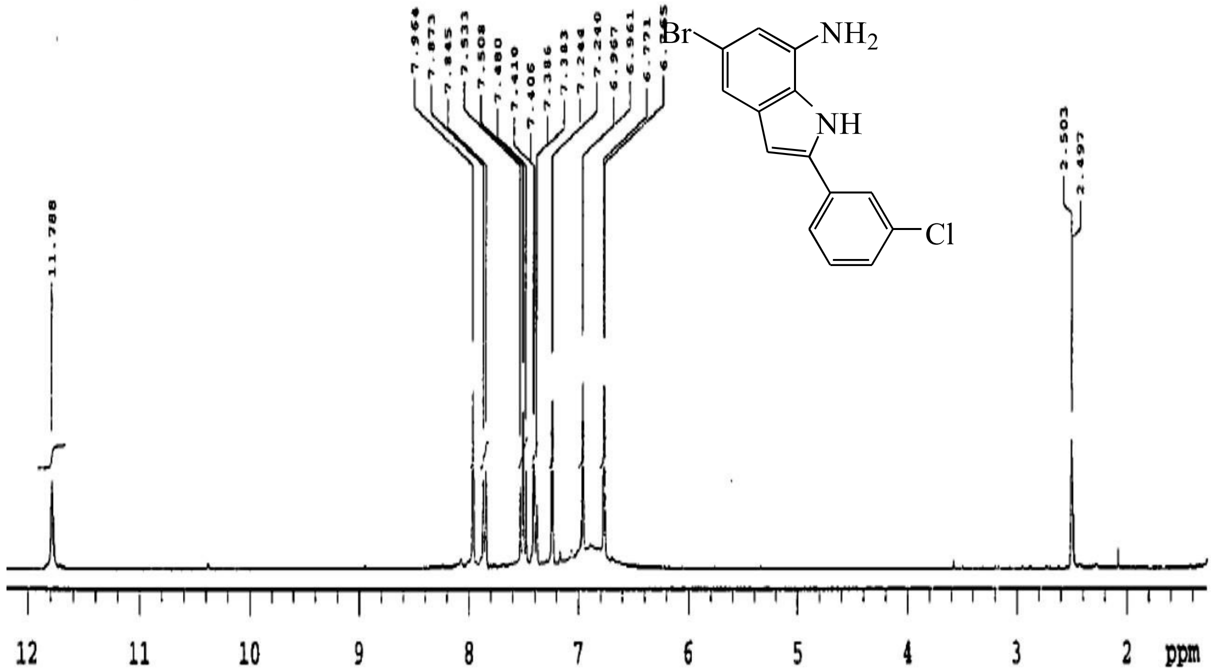

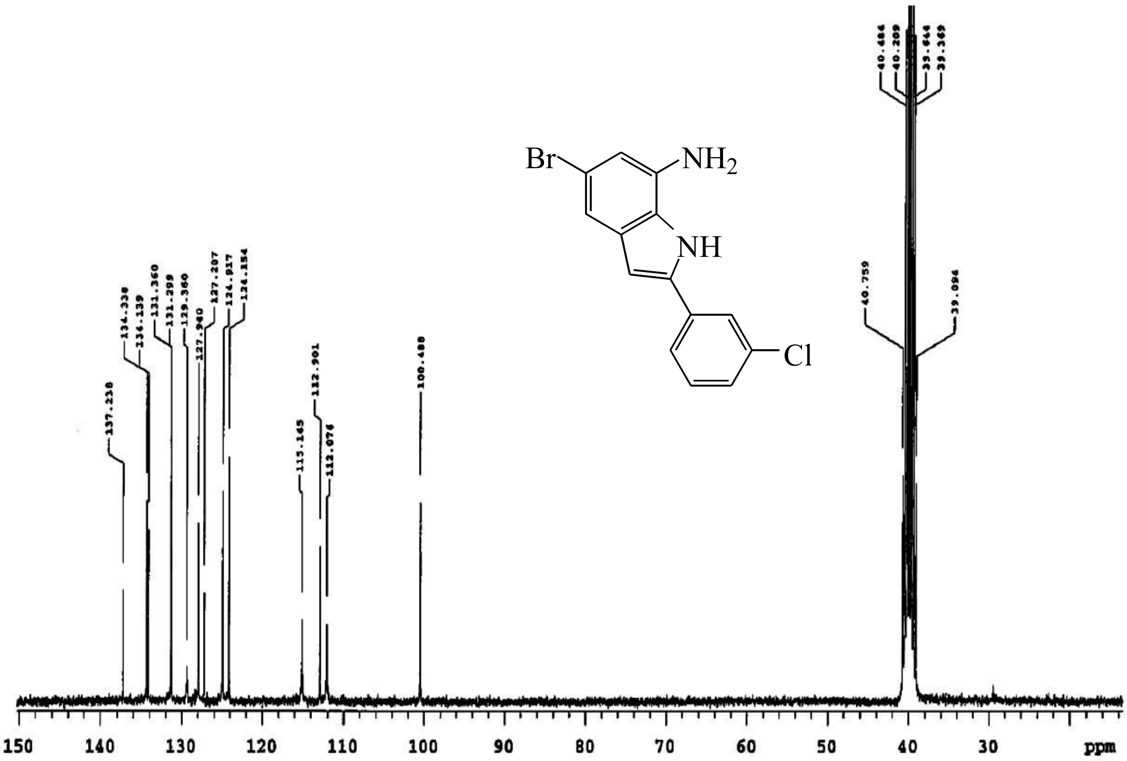

Figure S1.3: ^{1}H- and ^{13}C-NMR spectra of **2c** in DMSO-d_6 at 300 MHz and 75 MHz, respectively

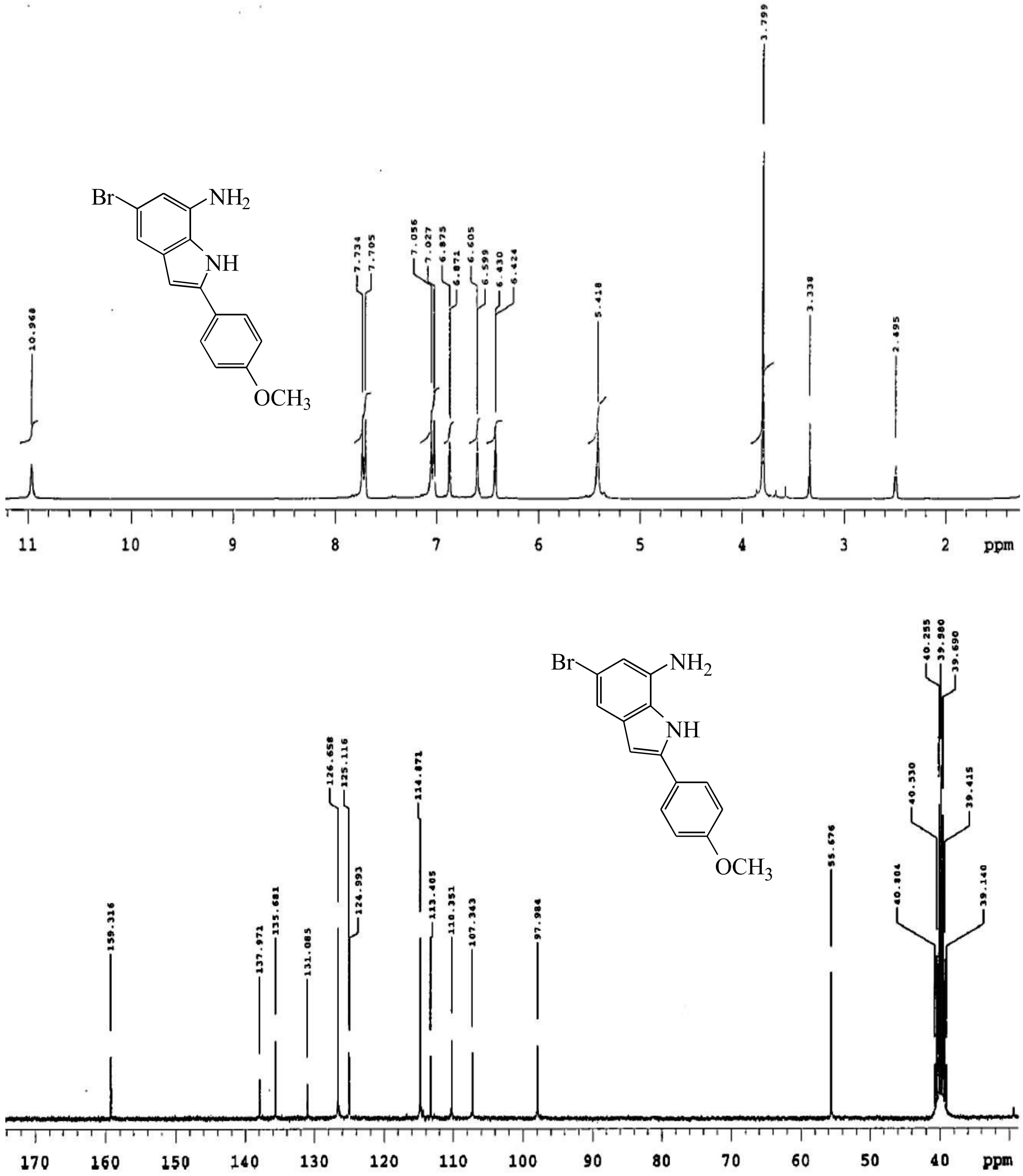

Figure S1.4: ^{1}H- and ^{13}C-NMR spectra of **2d** in DMSO-d_6 at 300 MHz and 75 MHz, respectively

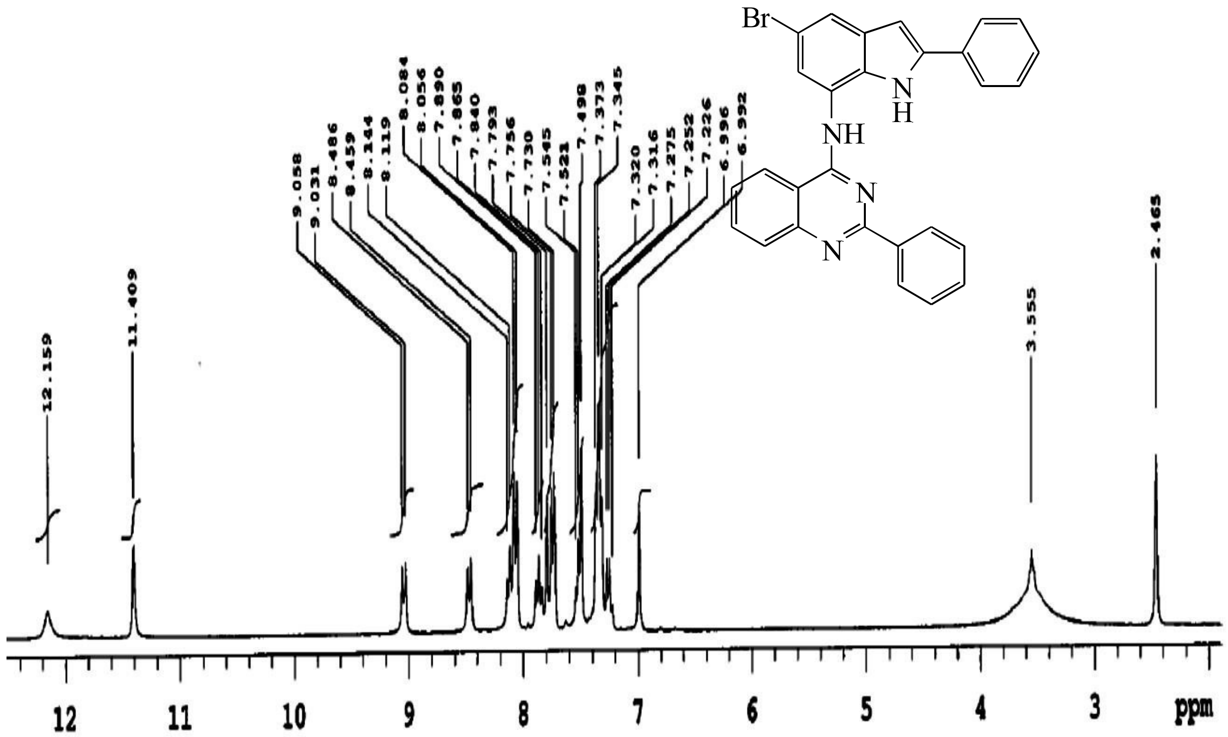
Br
N
H
NH
N
N
12.159
11.409
9.058
9.031
8.486
8.459
8.144
8.119
8.084
8.056
7.890
7.865
7.840
7.793
7.756
7.730
7.545
7.521
7.498
7.373
7.345
7.320
7.316
7.275
7.252
7.226
6.996
6.992
3.555
2.465
12
11
10
9
8
7
6
5
4
3
ppm

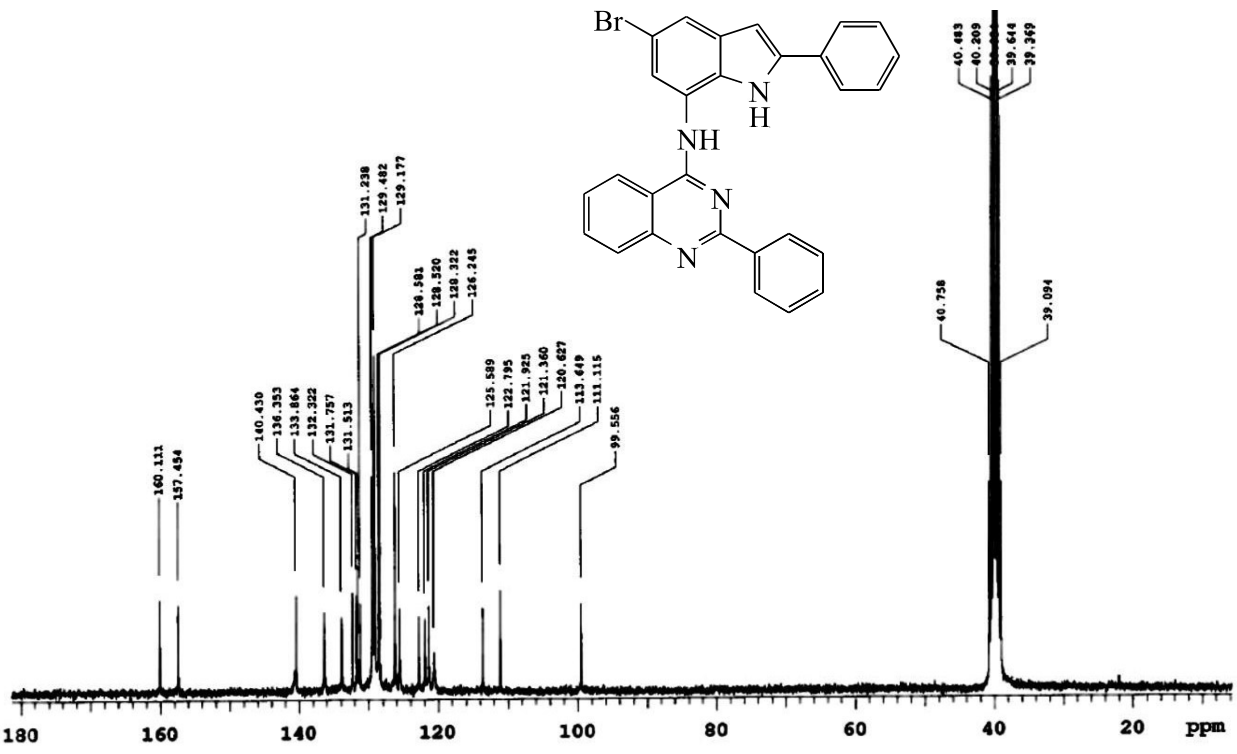
Br
N
H
NH
N
N
160.111
157.454
140.430
136.353
133.864
132.322
131.757
131.513
131.238
129.482
129.177
128.581
128.520
128.322
126.245
125.589
122.795
121.925
121.360
120.627
113.649
111.115
99.556
40.483
40.209
39.644
39.369
40.758
39.094
180
160
140
120
100
80
60
40
20
ppm

Figure S1.5: ^{1}H- and ^{13}C-NMR spectra of **4a** in DMSO-d_6 at 300 MHz and 75 MHz, respectively

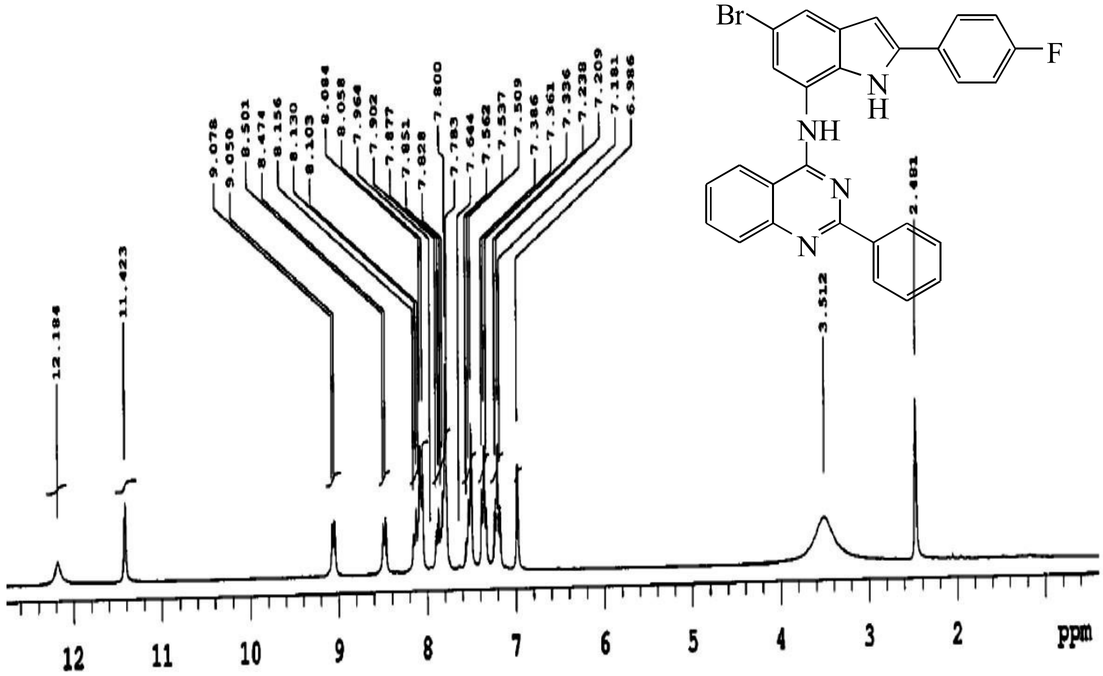

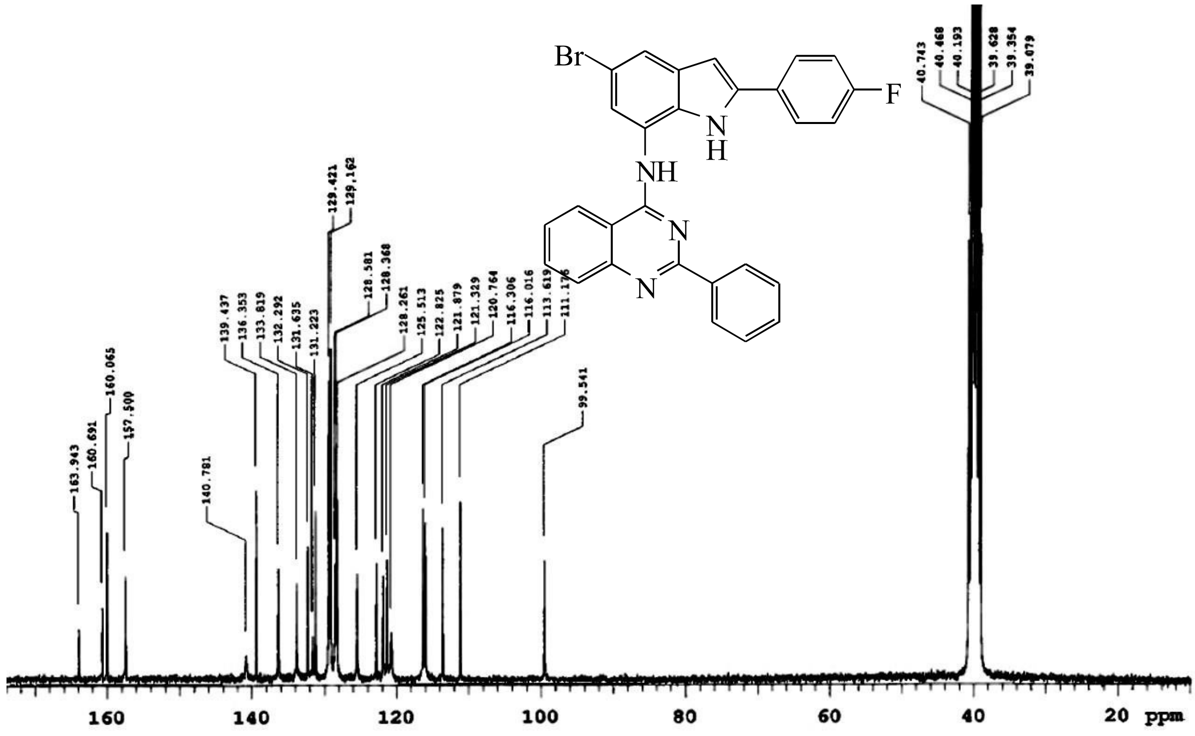

Figure S1.6: ^{1}H- and ^{13}C-NMR spectra of **4b** in DMSO-d_6 at 300 MHz and 75 MHz, respectively

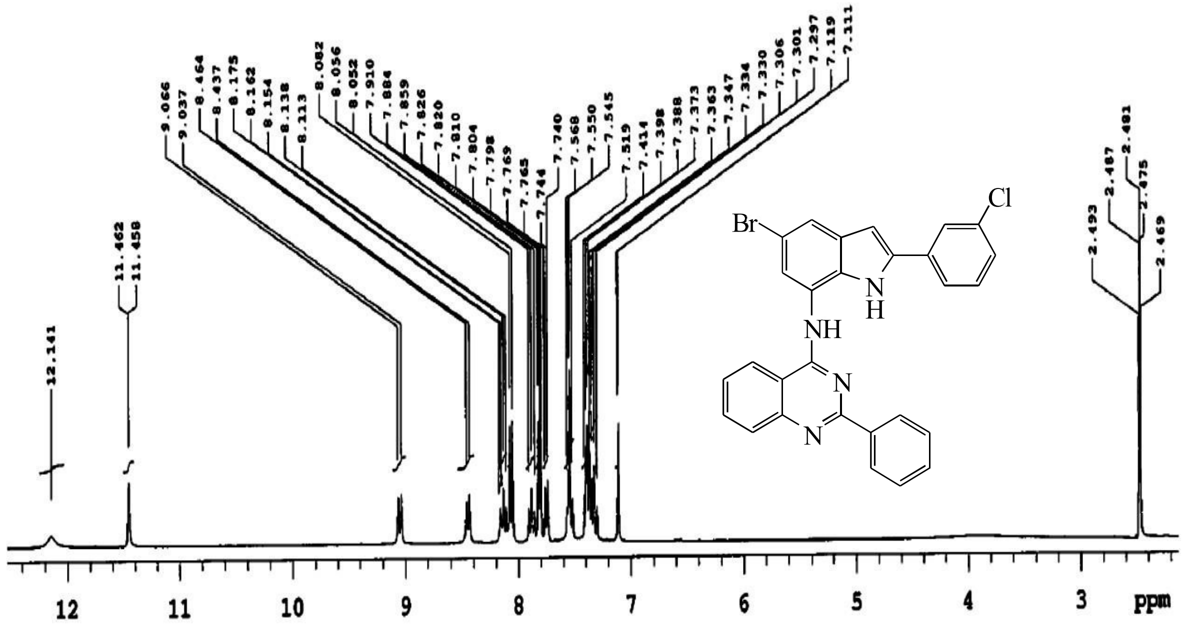

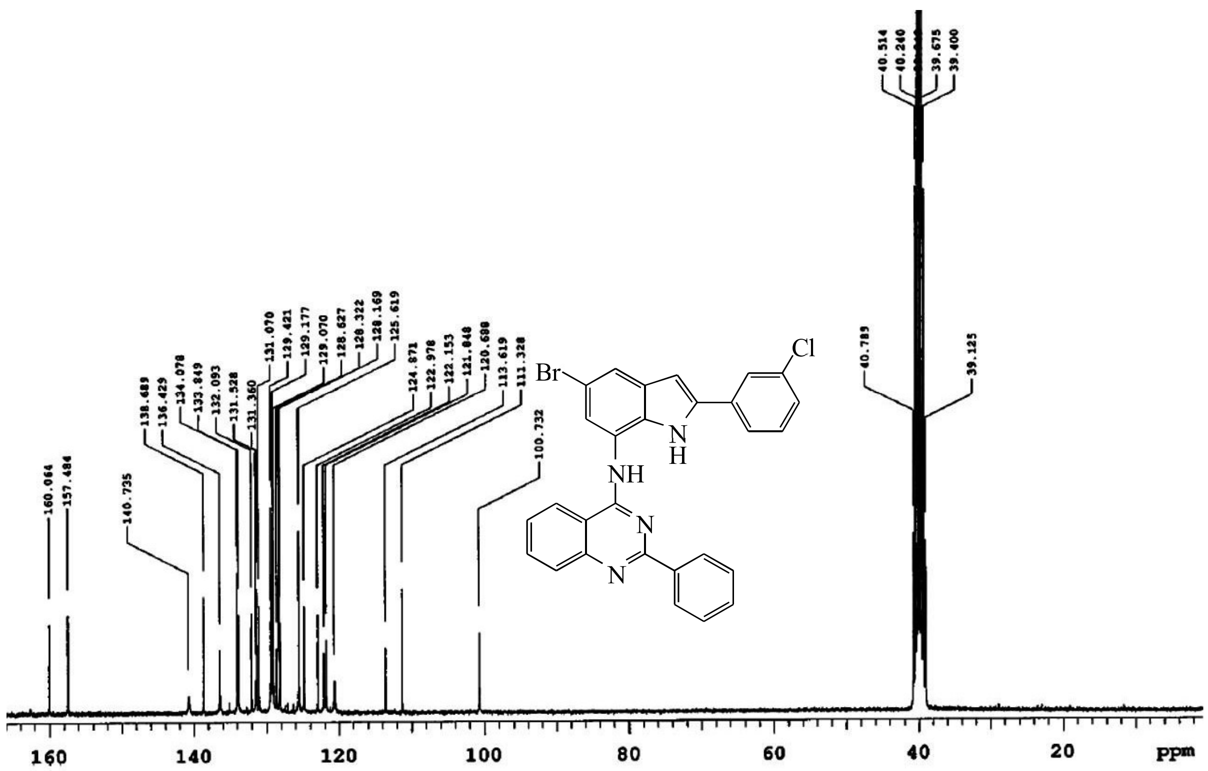

Figure S1.7: ¹H- and ¹³C-NMR spectra of **4c** in DMSO-d_6 at 300 MHz and 75 MHz, respectively

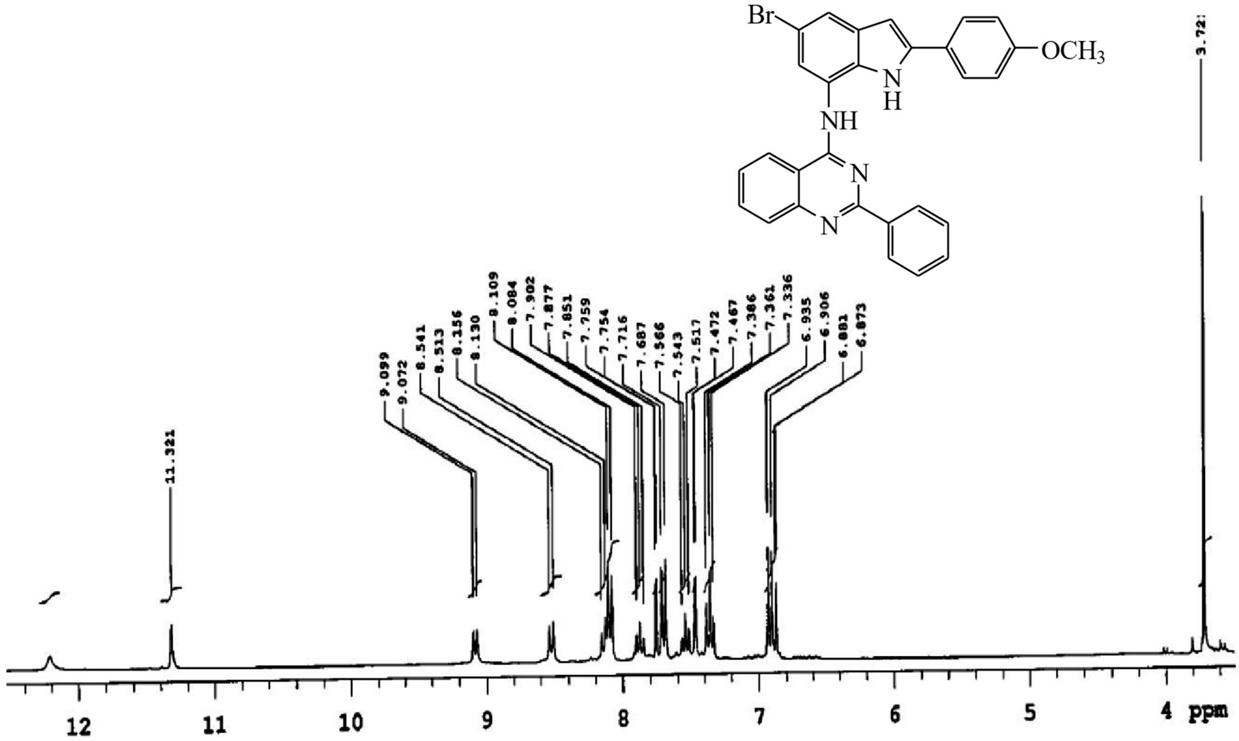

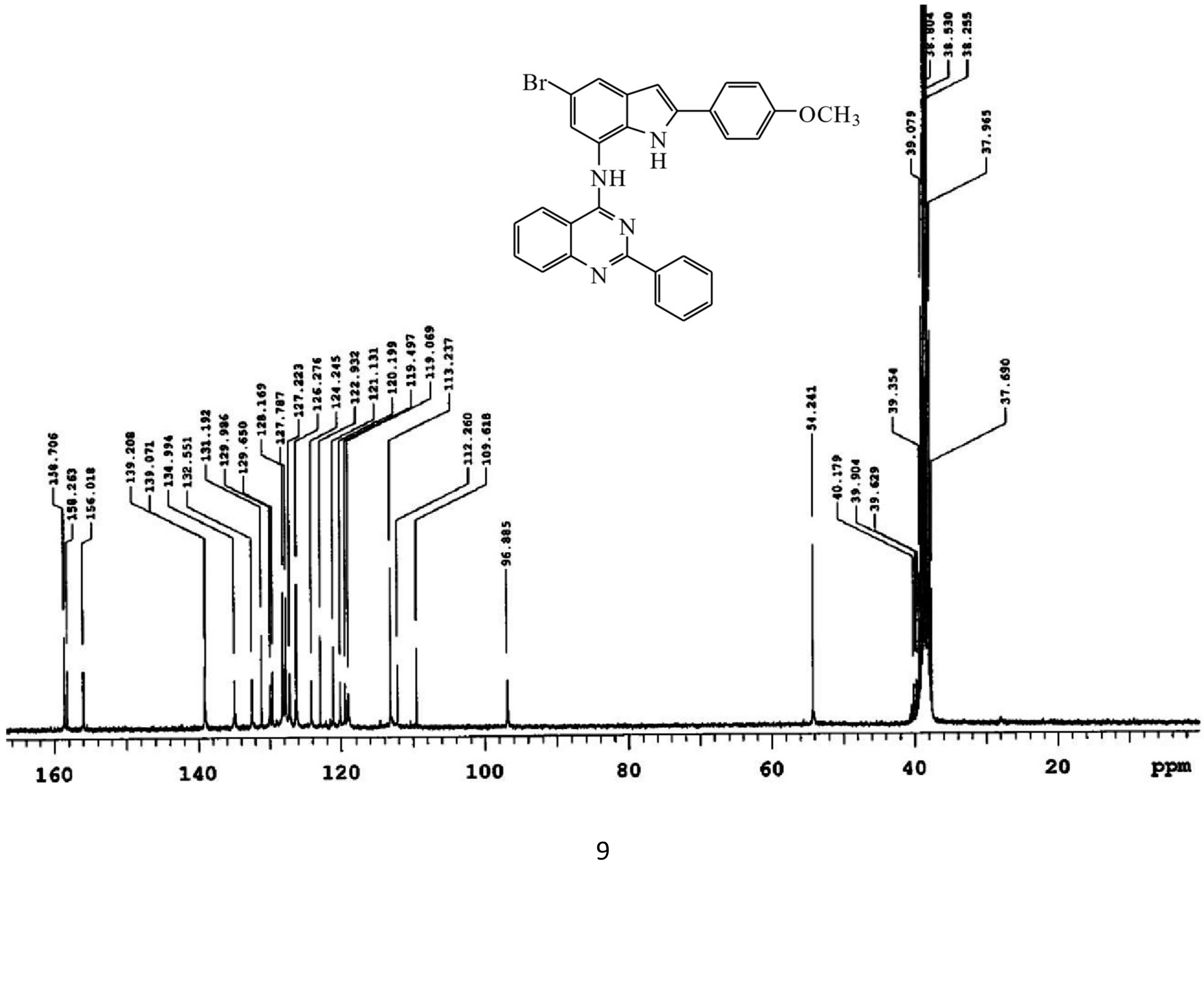

Figure S1.8: ^{1}H- and ^{13}C-NMR spectra of **4d** in DMSO-d_6 at 300 MHz and 75 MHz, respectively

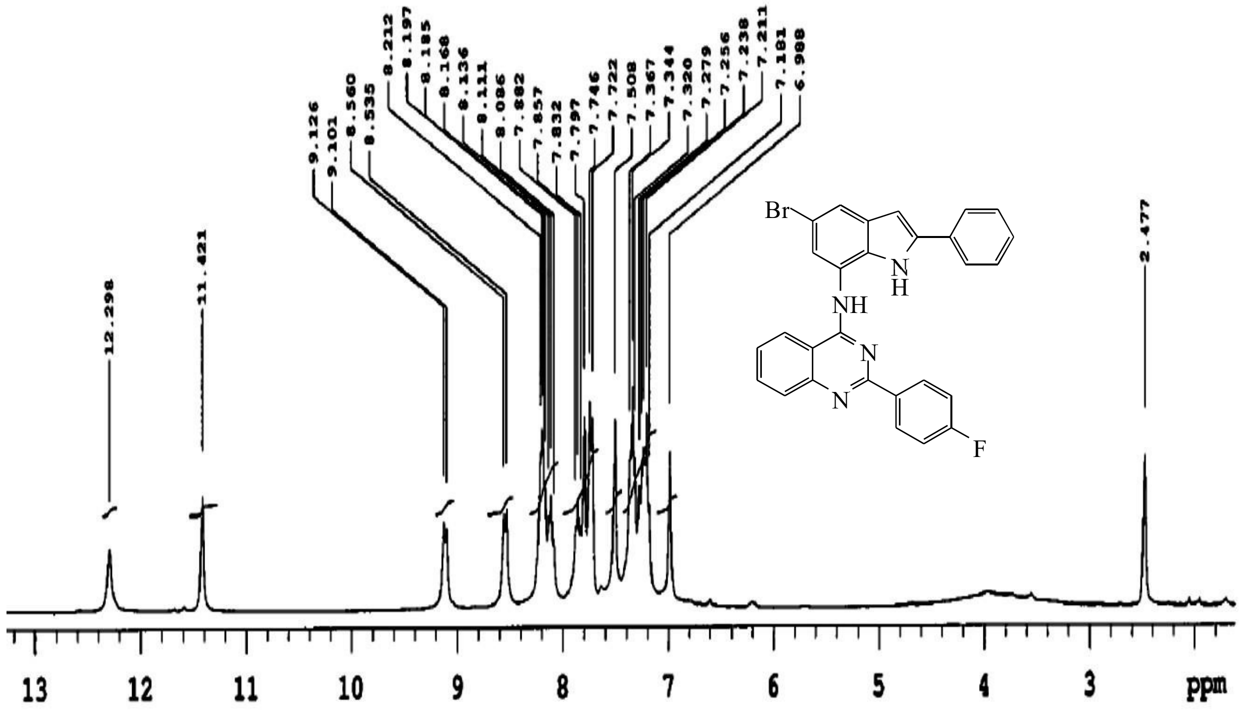

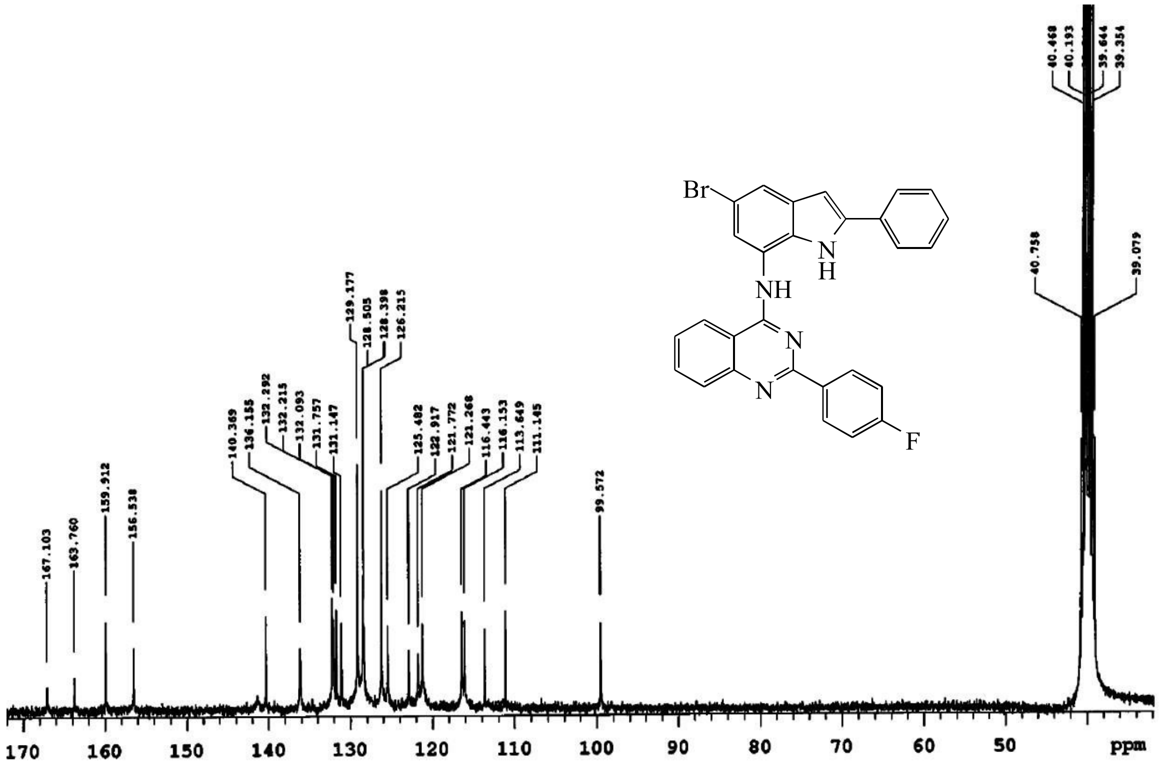

Figure S1.9: ^{1}H- and ^{13}C-NMR spectra of **4e** in DMSO-d_6 at 300 MHz and 75 MHz, respectively

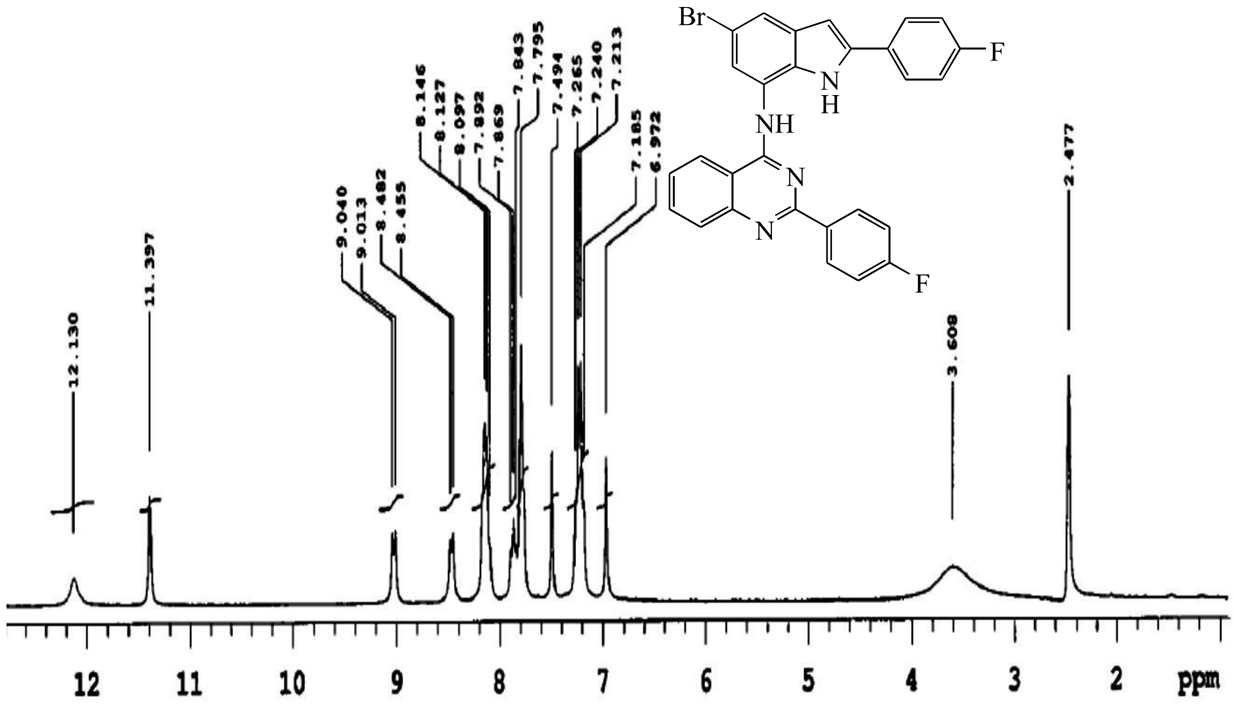

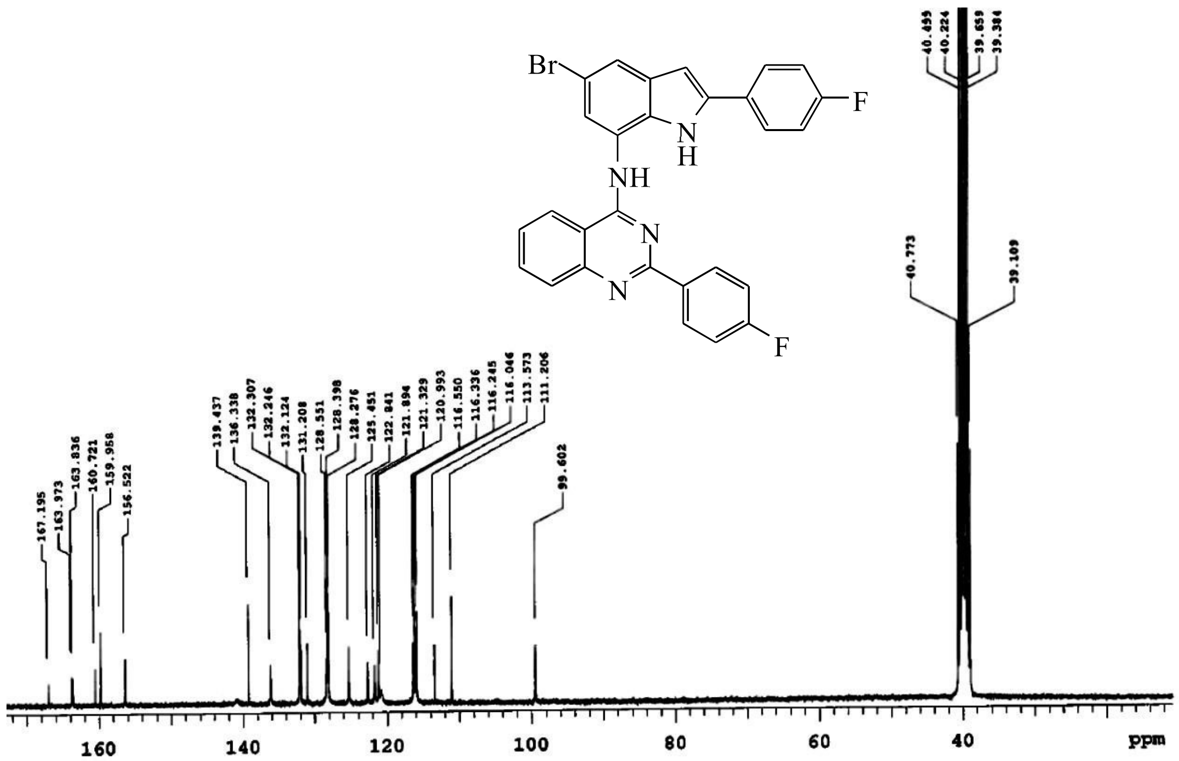

Figure S1.10: ^{1}H- and ^{13}C-NMR spectra of **4f** in DMSO-d_6 at 300 MHz and 75 MHz, respectively

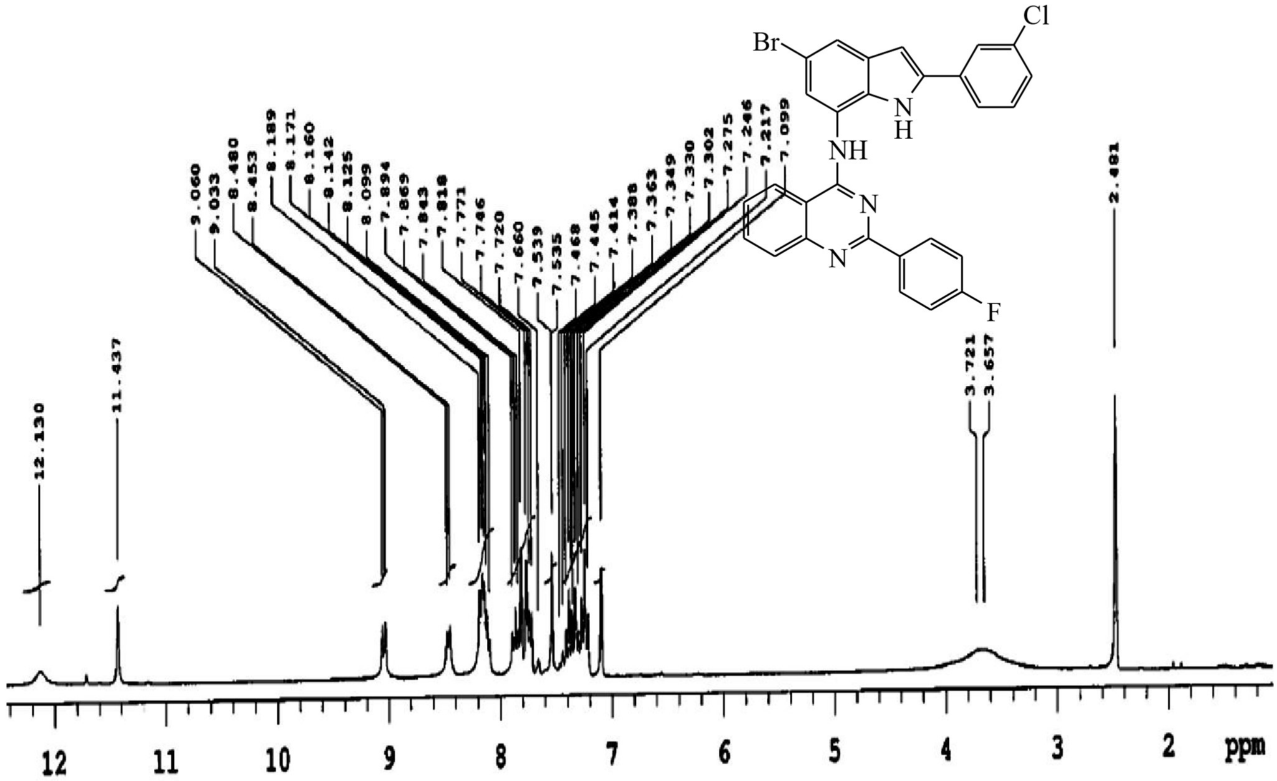
Br
Cl
N
H
NH
N
N
F
12.130
11.437
9.060
9.033
8.480
8.453
8.189
8.171
8.160
8.142
8.125
8.099
7.894
7.869
7.843
7.818
7.771
7.746
7.720
7.660
7.539
7.535
7.468
7.445
7.414
7.388
7.363
7.349
7.330
7.302
7.275
7.246
7.217
7.099
3.721
3.657
2.481
12 11 10 9 8 7 6 5 4 3 2 ppm

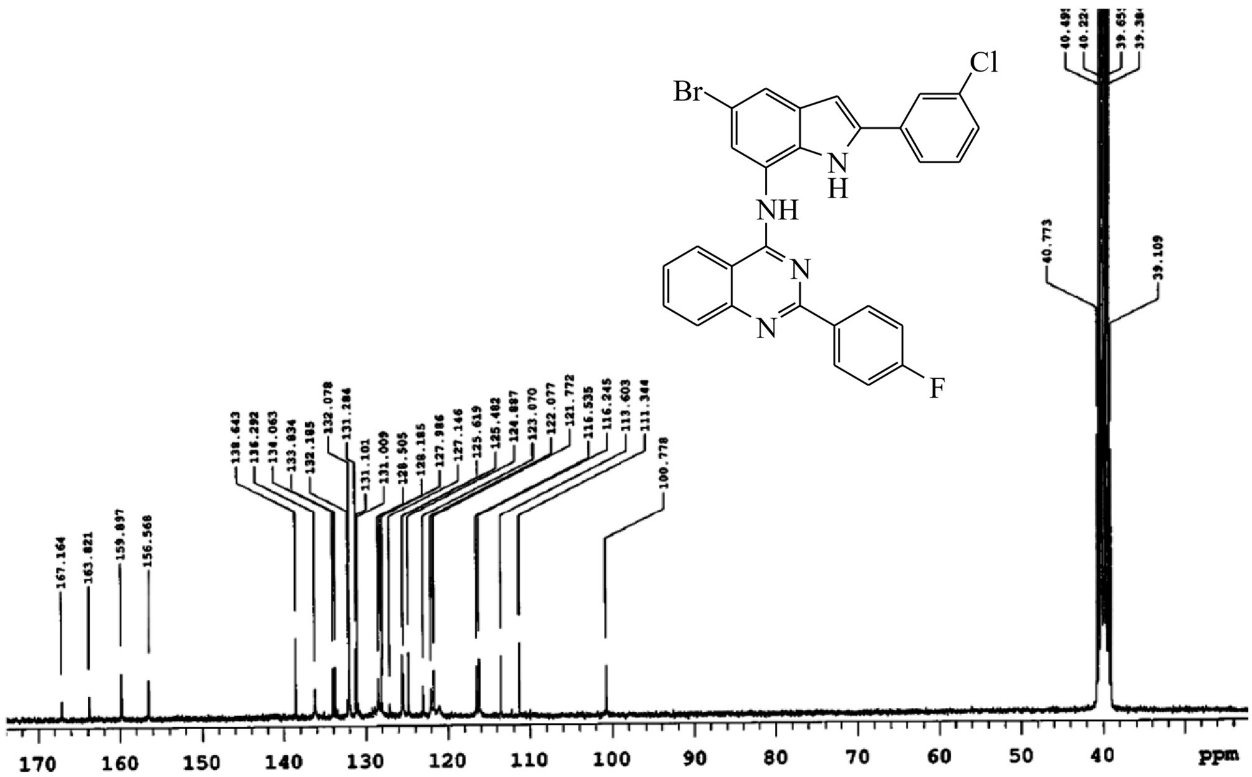
Br
Cl
N
H
NH
N
N
F
167.164
163.821
159.897
156.568
138.643
136.292
134.063
133.834
132.185
132.078
131.284
131.101
131.009
128.505
128.185
127.986
127.146
125.619
125.482
124.887
123.070
122.077
121.772
116.535
116.245
113.603
111.344
100.778
40.773
39.109
170 160 150 140 130 120 110 100 90 80 70 60 50 40 ppm

Figure S1.11: ^{1}H- and ^{13}C-NMR spectra of **4g** in DMSO-*d₆* at 300 MHz and 75 MHz, respectively

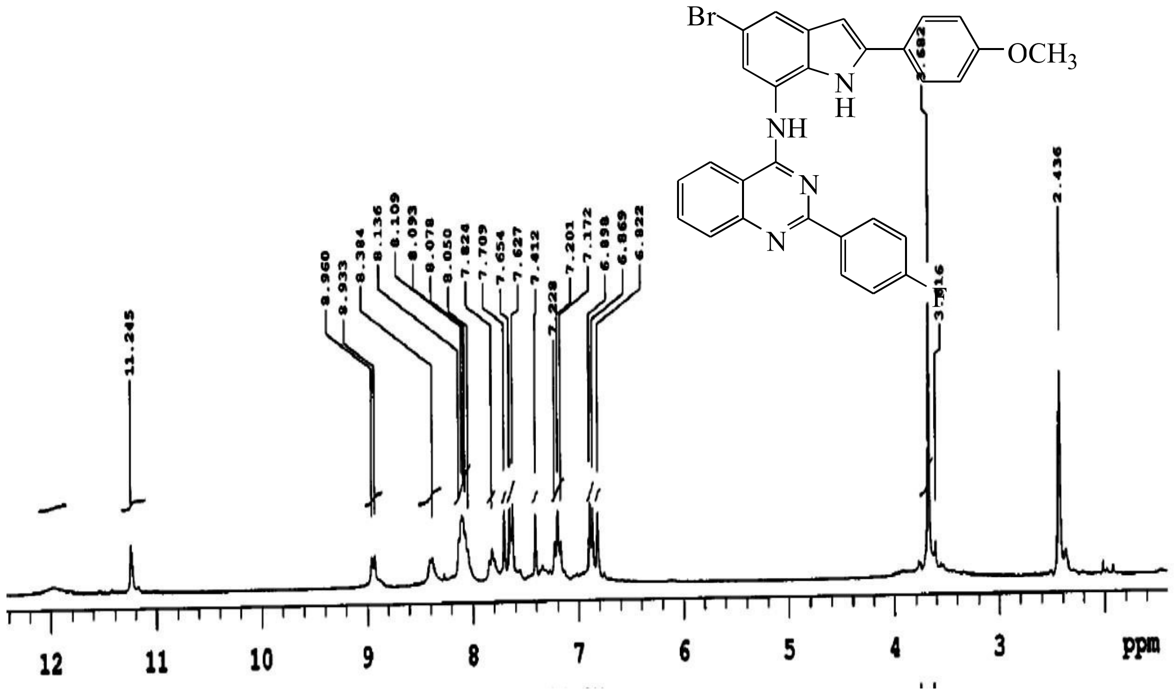

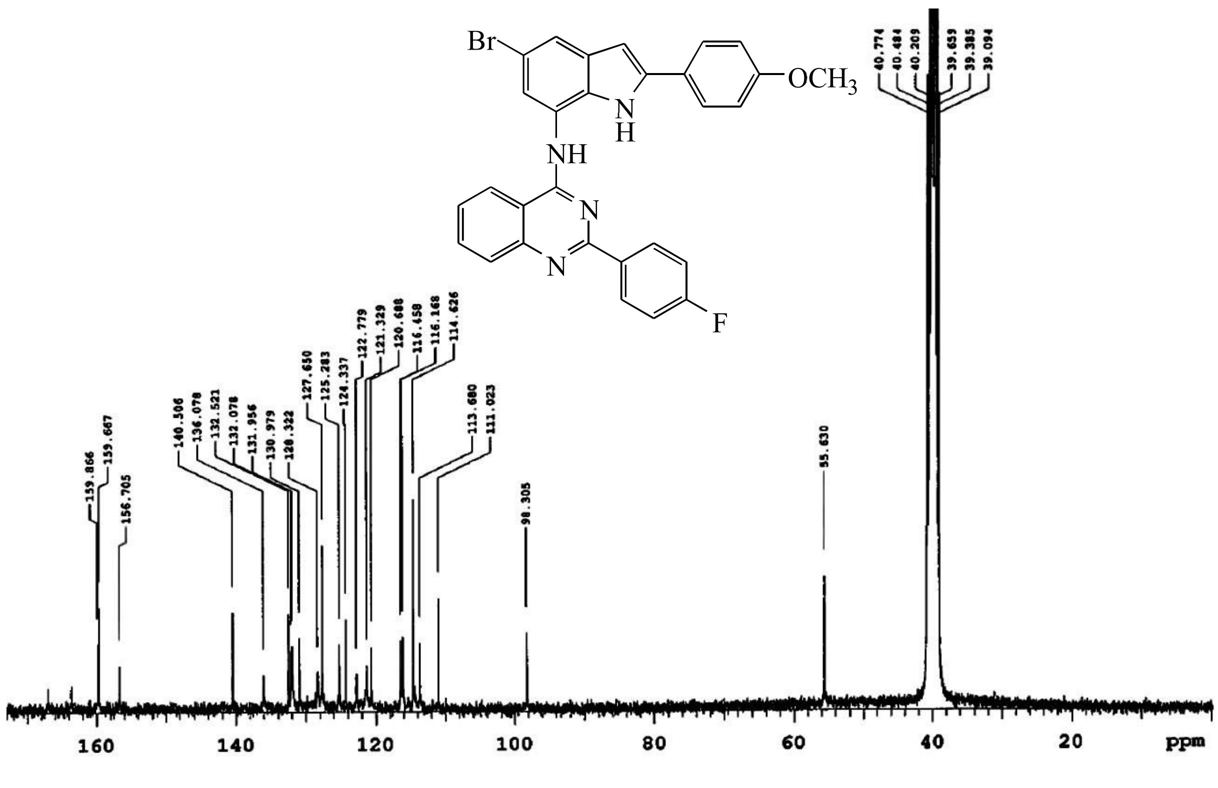

Figure S2: Docked conformation of erlotinib, gefitinib, indole-aminoquinazolines **4a–h**

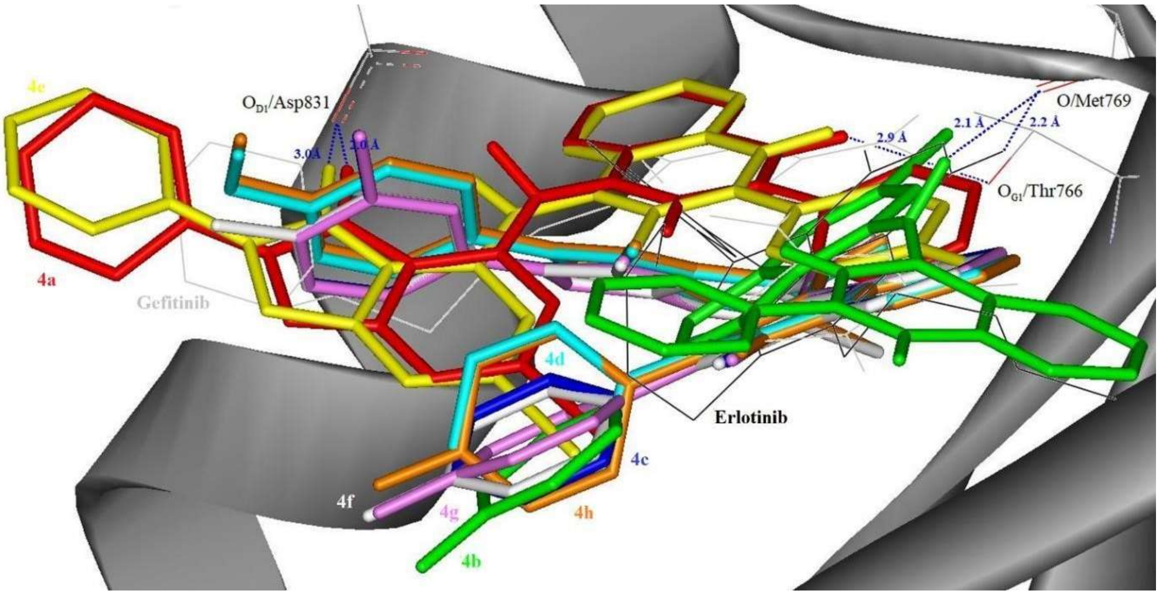

Figure S2: Docked conformation of erlotinib (as control), gefitinib, indole-aminoquinazolines **4a–h** (stick representation) in the binding pocket of the EGFR kinase domain (surface and ribbon representation). Blue dotted lines indicate the direct hydrogen bonding formed between the docked ligand and EGFR.
